# Superframe Duration Allocation Schemes to Improve the Throughput of Cluster-Tree Wireless Sensor Networks

**DOI:** 10.3390/s17020249

**Published:** 2017-01-27

**Authors:** Erico Leão, Carlos Montez, Ricardo Moraes, Paulo Portugal, Francisco Vasques

**Affiliations:** 1 INEGI/INESC-TEC, Faculty of Engineering, University of Porto, 4200-465 Porto, Portugal; pportugal@fe.up.pt (P.P.); vasques@fe.up.pt (F.V.); 2 Department of Computing, Federal University of Piauí, 64049-550 Teresina, Brazil; 3 Department of Automation and Systems, Federal University of Santa Catarina, 88040-900 Florianópolis, Brazil; carlos.montez@ufsc.br; 4 Department of Computing, Federal University of Santa Catarina, 88905-120 Araranguá, Brazil; ricardo.moraes@ufsc.br

**Keywords:** IEEE 802.15.4, ZigBee, cluster-tree, wide-scale, allocation scheme, superframe duration, congestion, buffer overflow

## Abstract

The use of Wireless Sensor Network (WSN) technologies is an attractive option to support wide-scale monitoring applications, such as the ones that can be found in precision agriculture, environmental monitoring and industrial automation. The IEEE 802.15.4/ZigBee cluster-tree topology is a suitable topology to build wide-scale WSNs. Despite some of its known advantages, including timing synchronisation and duty-cycle operation, cluster-tree networks may suffer from severe network congestion problems due to the convergecast pattern of its communication traffic. Therefore, the careful adjustment of transmission opportunities (superframe durations) allocated to the cluster-heads is an important research issue. This paper proposes a set of proportional Superframe Duration Allocation (SDA) schemes, based on well-defined protocol and timing models, and on the message load imposed by child nodes (*Load-SDA* scheme), or by number of descendant nodes (*Nodes-SDA* scheme) of each cluster-head. The underlying reasoning is to adequately allocate transmission opportunities (superframe durations) and parametrize buffer sizes, in order to improve the network throughput and avoid typical problems, such as: network congestion, high end-to-end communication delays and discarded messages due to buffer overflows. Simulation assessments show how proposed allocation schemes may clearly improve the operation of wide-scale cluster-tree networks.

## 1. Introduction

With the increasing technological advances of Micro-Electro-Mechanical devices [1], including its processing and storing capabilities, Wireless Sensor Networks (WSN) have become an attractive technology to deploy wide-scale applications, such as: environmental monitoring, precision agriculture, smart buildings and cities, industrial automation and military [2,3].

WSNs are special wireless *ad hoc* networks composed of a large number of low-power, low-cost and low-rate devices, which are capable of sensing, processing and sending information related to environment variables [4]. This type of network may also be able to actuate over the monitored environment, through the use of special devices called actuators. The increasing demand for WSN-based applications is driving the need for new design approaches, able to deal with WSN specific requirements, such as energy-efficient operation, wide-scale deployments and time-sensitive approaches.

IEEE (Institute of Electrical and Electronics Engineers) 802.15.4 standard [5] and ZigBee specification [6] define the most widely used protocols to deploy WSNs. On the one hand, IEEE 802.15.4 standard defines the Physical layer and Medium Access Control (MAC) sublayer for Low-Rate Wireless Personal Area Network (LR-WPAN) applications. On the other hand, ZigBee specifies the upper layers (Network and Application) for the IEEE 802.15.4 protocol stack. Basically, this set of documents defines two types of devices: Full-Function Devices (FFD) and Reduced-Function Devices (RFD). While RFDs perform only a reduced set of functions such as channel scanning, network association requests and sensing activities, FFDs can also perform more complex functions, such as PAN and cluster coordination, routing, forwarding, and packet aggregation or fusion.

Depending on the application requirements, an IEEE 802.15.4 network can operate according to two topologies: star and peer-to-peer. The star topology is the simplest network organisation, where all sensing nodes are directly connected to the PAN coordinator. This PAN coordinator is a unique node, that is responsible for all management and communication activities (centralised communication paradigm). Although being easy to build and manage, the weakness of this topology is that its coverage is limited by the sensing range of its nodes, which prevents wide-scale deployments.

In peer-to-peer topologies, any device may directly communicate with any other device, as long as they are in the communication range of each other. This topology allows more complex network formations, such as cluster-tree and mesh topologies. Mesh topologies provide higher network flexibility and lower complexity, high routing redundancy and good network coverage [7]. However, it does not provide explicit mechanisms allowing nodes to enter in low power mode [8,9], decreasing the network lifetime. These characteristics are not desirable for typical WSN-based monitoring applications, which impose strict requirements regarding the power consumption of sensor nodes.

In order to overcome this weakness, the IEEE 802.15.4/ZigBee set of specifications also provides the cluster-tree topology—a special peer-to-peer topology. Cluster-tree is one of the most suitable topologies to deploy wide-scale WSNs [10]. In the cluster-tree topology, nodes are grouped into clusters and coordinated by a unique FFD node called cluster-head (the PAN coordinator is a specific case of a cluster-head). Cluster-heads are responsible for association, synchronisation and communication of their child nodes. In order to provide scalability, clusters are interconnected through their coordinators building a hierarchical network structure.

Nevertheless, the efficient operation of cluster-tree topologies requires the consideration of some relevant design issues, such as: network formation [11,12], beacon scheduling [13,14], and MAC protocol configuration issues [15,16,17,18], including those related to the medium access protocol and the definition of communication structures. Thus, it is necessary to provide adequate guidelines for setting-up the MAC configuration parameters, in order to build efficient wide-scale cluster-tree WSNs.

### 1.1. Objectives and Contributions of This Paper

In this paper, we define a holistic approach to deal with the problem of how to allocate superframe durations to multiple cluster-heads in a wide-scale WSN. This paper extends the work previously presented in [19], where it was defined a simple protocol constraint for cluster-tree networks and proposed a superframe allocation scheme based on the traffic load generated by sensor nodes. We define a set of boundary equations, that act as upper-bounds for the protocol operating behaviour of IEEE 802.15.4 cluster-tree networks. Based on these boundary equations, we propose a set of superframe duration allocation schemes to be applied at system design time (offline scheduling). These schemes are able to improve the network throughput in wide-scale WSN deployments. They distribute the available network bandwidth among the different network clusters, according to the associated traffic. As a consequence, they enable the reduction of some of the typical problems of cluster-tree networks, such as: network congestion around the PAN coordinator, high message communication delays and a high number of discarded messages due to buffer overflows. Basically, the proposed superframe duration allocation schemes consider: (1) the message load imposed by sensor nodes; (2) the number of descendant nodes of each cluster; and (3) the number of child nodes belonging to the cluster itself. This type of bandwidth allocation schemes is based on earlier work done for Fieldbus networks [20] and for FDDI networks [21] and, more recently, for FlexRay networks [22,23].

We focus our study on ZigBee-based cluster-tree topologies, due to some of its specific features, such as suitability to deploy wide-scale networks with energy-efficiency Quality of Service (QoS), which are common requirements for typical WSN monitoring applications. We envisage the use of the proposed allocation schemes in real world monitoring applications, such as those that can be found in precision agriculture and environmental monitoring. One of the major requirements of this type of applications is the capability to deal with periodic data traffic generated by a large number of widely deployed sensor nodes. In precision agriculture applications, WSN-based technologies are responsible for automating information collection processes about the crop production, enabling actuating strategies to maximise the crop yield and quality, and to optimise the environmental resources [24,25,26].

The main contributions of this paper can be summarised as follows:

- A set of boundary equations for the operating behaviour of IEEE 802.15.4 cluster-tree networks, defining a set of constraints that must be fulfilled to guarantee the adequate timing behaviour of supported applications.
- A set of guidelines to define transmission opportunities (superframe durations) and buffer sizes for each cluster, in a cluster-tree network. These guidelines are based on both the traffic requirements and the network topology, including the number of child nodes and the depth of the cluster-heads in the cluster-tree network. The use of such guidelines significantly increases the network throughput, avoiding or reducing the congestion of the cluster-tree network.
- A simulation assessment that highlights the advantages of using the proposed superframe duration allocation schemes for wide-scale deployments of wireless sensor networks.

### 1.2. Organisation of This Paper

The remainder of this paper is organised as follows: Section 2 provides the required background. Section 2.1 presents an overview of IEEE 802.15.4/ZigBee cluster-tree networks and Section 2.2 presents some of the most relevant related works for the development of this proposal. Section 3 defines the considered message traffic and network models. Section 4 presents a set of boundary equations that constrain the allocation of superframe durations for each cluster-head. Section 5 models the considered message transmission duration time. Section 6 presents a timing constraint for the monitoring traffic, based on the protocol constraints of IEEE 802.15.4 cluster-tree networks. Section 7 introduces the proposed superframe duration allocation schemes. Section 7.1 presents an allocation scheme based on the load imposed by the descendant nodes of each cluster-head; Section 7.2 presents an allocation scheme based on the number of descendant nodes; Section 7.3 provides an example of the use of the proposed allocation schemes. Finally, Section 8 presents a simulation assessment of the proposed allocation schemes and discussion of the results, and some conclusions and considerations about future works are presented in Section 9.

## 2. Background

### 2.1. Cluster-Tree Topologies

Typical WSNs application deployments have multiple sensor nodes and one coordinator, generally located in the middle of the topology (star topology). A common way to achieve scalability is to connect multiple star networks, resulting in cluster-tree topologies that are complex peer-to-peer constructions, where sensor nodes are grouped into clusters. Each cluster is coordinated by a specific FFD node called coordinator or cluster-head (CH). All communication within the clusters is centralised under the control of the CH. The CH is responsible for building its own cluster, managing nodes’ association and providing synchronisation mechanisms and intra-cluster communication.

The first cluster of the network is built by a special node, called PAN coordinator. The PAN coordinator can be considered a special node with a powerful central processing unit, being responsible for all the network management activities and is usually the sink node of the network. The CHs (including the PAN coordinator) are interconnected by parent-child relationships, forming a hierarchical network structure (multicluster).

**Figure 1:**
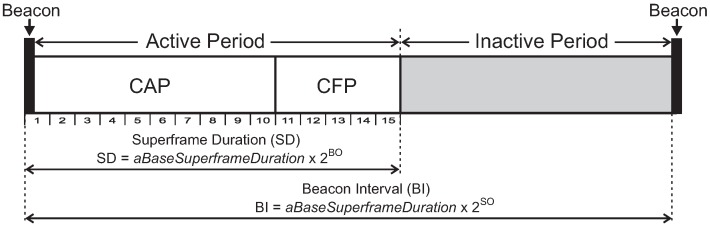
IEEE 802.15.4 Superframe structure.

Cluster-tree networks operate in a beacon-enabled mode, where a structure called *Superframe* organises all communication rounds. A superframe is bounded by beacon frames, which are periodically transmitted by cluster-heads. Beacon frames are used to synchronise clusters and also to describe the superframe structure. This structure is described by two parameters: the *macBeaconOrder* (BO) and *macSuperframeOrder* (SO), where 0≤SO≤BO≤14. These parameters define the *Beacon Interval* (BI) and the *Superframe Duration* (SD), respectively. Figure 1 illustrates the superframe structure.

BI defines the periodicity at which cluster-heads must transmit their beacon frames. In turn, SD defines the communication period of the clusters. Each superframe can be composed of two parts: active and inactive periods. The inactive period exists only if the SO parameter is smaller than the BO parameter. During the active period, nodes can communicate with their cluster-heads. On the other hand, during inactive period, the coordinator and member nodes may enter in low-power (sleep) mode in order to save energy. The active part comprises two periods: *Contention Access Period* (CAP), during which member nodes can communicate using a slotted *Carrier Sense Multiple Access with Collision Avoidance* (CSMA-CA) mechanism to access the channel; and *Contention-Free Period* (CFP), during which the coordinator can allocate up to seven *Guaranteed Time Slots* (GTS) for specific devices to transmit data without contending for the channel access. The *aBaseSuperframeDuration* parameter defines the minimum duration of a superframe ($SD_{min}$) when SO is 0 (by default, this parameter corresponds to 960 symbols, corresponding to a duration of 15.36 ms, considering a bit rate of 250 kbps, frequency band of 2.4 GHz, and one symbol as 4 bits).

### 2.2. Related Work

In recent years, multiple research works have been presented addressing some of the most relevant challenges concerning cluster-tree WSNs, including network formation schemes, communication mechanisms, MAC protocol configuration, energy-efficiency, scalability, admission and congestion control, and beacon scheduling. Each of these issues has its own special considerations. Within the context of this paper, we are particularly interested in works that address throughput in IEEE 802.15.4 cluster-tree WSNs, using CAP communication mechanisms. For this topic, modelling the main constraints of the IEEE 802.15.4 MAC protocol and defining adequate communication periods are important mechanisms to avoid known problems such as packet drops due to buffer overflows, network congestion and high end-to-end communication delays, which may lead to undesired operation of the network [27]. Several works in the literature show that the configuration of IEEE 802.15.4 MAC parameters has a direct impact on the performance of WSNs, in what concerns energy efficiency, wide-scale deployments and time-sensitive message transfers [15,18].

In this context, we point out a set of works [28,29,30,31] that provide analytical models for the timing analysis of the contention access period of the IEEE 802.15.4 MAC protocol. Cao et al. [28] present an accurate analytical model to evaluate the behaviour of the IEEE 802.15.4 MAC protocol with periodic traffic, which is a common scenario for WSN-based monitoring applications. The authors consider the probabilities of CCA (Clear Channel Assessment) failures and transmission collisions, considering the standard characteristics of retransmissions and the double CCA of CSMA-CA protocol. Although the authors point out that this model can be used to define adequate active period durations, no scheme or guidelines are provided.

Several works encompass analytical models for the contention period of IEEE 802.15.4 MAC protocol, based on Markov chains [29,30,31]. Basically, each of these analytical models considers a specific set of parameters and characteristics of the CSMA-CA protocol. Guennoun et al. [29] provide a new IEEE 802.15.4-based MAC protocol named *Variable CCA MAC protocol*. The idea behind this proposal is to change the number of CCAs that a node must perform before transmitting a data packet (by default, this parameter is defined as 2). The authors model this new protocol using Markov chains and demonstrate its accuracy and capability of predicting its behaviour through simulation. However, this new protocol has a negative performance regarding channel utilisation, communication delays, reliability and energy consumption. In fact, increasing the number of CCAs leads to the increase of the contention window, which can generate higher delays and energy consumption. Furthermore, performing a higher number of CCAs does not avoid collisions, as nodes are not linearly spread along the time. Recas et al. [30] proposed an analytical model based on Markov chains, considering several node classes, by setting different values for the CSMA-CA protocol, but without considering inactive periods. Instead, Park et al. [31] provided two Markov chains to model the behaviour of IEEE 802.15.4 MAC protocol, considering both CAP and CFP. This analytical model considers the main parameters of CSMA-CA protocol.

A major drawback of these approaches is that they do only consider star networks and do not address the characteristics of cluster-tree networks. Thus, they are limited to specific environments and can not be applied to wide-scale applications. In addition, other weakness of these works is that they provide analytical models for the IEEE 802.15.4 MAC protocol without considering any scheme to adequately allocate the active period durations, in order to improve the network throughput.

Other works addressed analytical models encompassing cluster-tree networks [32,33,34]. Martalò et al. [32] proposed an analytical framework to model the behaviour of the IEEE 802.15.4 MAC protocol based on Markov chains. An important requirement assumed by the authors is the finite buffer queues for the nodes. The proposed model is quite simple, where the node traffic is always generated by nodes and forwarded toward the sink node (PAN coordinator), but it does not describe the complex features of this network type. Jurcík et al. [33] used network calculus theory to model cluster-tree WSNs according to several network parameters, such as: depth, maximum number of child nodes and maximum number of child routers. In that work, the authors provide a worst-case behaviour evaluation for upstream and downstream data flows, considering important constraints such as: buffer and bandwidth requirements, flow directions and end-to-end communication delays. However, they consider only contention-free periods (GTS), where the limitation of a maximum of seven GTS restricts the number of data flows in the network. Moreover, data traffic generated by typical monitoring scenarios was not considered in this analysis. Kohvakka et al. [34] provided several mathematical models for the timing analysis of IEEE 802.15.4 CSMA-CA mechanisms and MAC operation and verified the proposed models through simulations. However, only models were provided and no further schemes were proposed.

Moreover, we highlight other set of works presented by [9,35,36], which provide performance assessments of the MAC protocol by setting different values for its parameters. Di francesco et al. [9] analysed the impact of MAC parameters upon the network communication behaviour and proposed an adaptive cross-layer framework to minimise the energy consumption for single and multihop WSNs. The authors show that changing the *macMinBE*, *macMaxCSMABackoffs* and *macMaxFrameRetries* parameters may increase the probability of winning the contention for the wireless channel. However, this approach is valid just up to a certain threshold. They also show that *macMaxCSMABackoff* parameter has a higher impact over the energy consumption than *macMinBE* parameter. Severino et al. [35] proposed the TRADIF methodology, which enables traffic differentiation during the contention-access period, by setting different values of CSMA-CA parameters for critical messages (such as alarm reports and management messages), in order to provide higher priority and quality-of-service for this type of traffic. Chen et al. [36] provided a performance evaluation of IEEE 802.15.4 star networks through simulation. Differently of [9], authors focused on the selection of SO and BO parameters and their impact upon different industrial scenarios. However, only star topologies were evaluated and no allocation schemes were proposed.

Some works have used other techniques to increase the bandwidth for message streams, such as: beacon scheduling [14,37,38] and superframe duration adjustment schemes [15,39,40]. Hanzalek and Jurcík [14] presented a Time-Division Cluster Scheduling (TDCS) mechanism to meet end-to-end deadlines of time-bounded message streams. This mechanism employs a pure time-division scheduling approach, avoiding the inter-cluster collision problem. The authors formulated the TCDS approach as a cyclic extension of the Resource Constrained Project Scheduling with Temporal Constraints (RCPS/TC), which defines a feasible schedule considering temporal and resource constraints for a set of tasks. After modelling this problem, they used an integer linear programming algorithm to solve the scheduling problem. Severino et al. [37] proposed a dynamic cluster scheduling scheme to provide QoS for different traffic flows in cluster-tree networks. In that work, the authors defined a run-time approach to re-order the involved clusters in specific message streams, considering their priorities, in order to minimise traffic latency. Also, this approach provides a mechanism to increase the size of superframe durations (bandwidth) of the involved clusters, using the global inactive period or the active periods of non-involved clusters. Yeh and Pan [38] proposed the Low-latency Two-way Beacon Scheduling (LTBS) approach for cluster-tree networks. In this approach, the authors modify the superframe structure to allow the broadcast of two beacons, defining one active part for the upstream traffic and another for downstream traffic. The authors also defined a set of algorithms to assign nodes to upstream and downstream slots in order to reduce the network latency, avoiding interferences among them.

In turn, Lee et al. [15] provided a Superframe Duration Adjustment Scheme (SUDAS) based on Markov chains, which analyses both the contention and contention-free periods, allocating GTS slots for devices based on the packet sizes. The underlying idea of SUDAS is to adequately allocate GTS for a set of requested devices, improving the bandwidth of the contention-free period. Rasouli et al. [39] proposed an algorithm for the adjustment of the superframe duration and the CSMA Backoff Exponent (BE) parameter according to the network traffic, in order to decrease energy consumption and to improve the network throughput. However, these schemes just consider star topologies. Casilari et al. [40] provided algorithms to define the superframe durations for all clusters in a cluster-tree network, following a time-division approach. The main idea is to maximise the use of the beacon interval and to avoid any inactive period. For this, the authors proposed different allocating schemes, such as: the same SO for all clusters, highest SO for the PAN coordinator and a scheme that allocates a SO for the coordinator based on the traffic generated in its cluster. However, the authors just present simple schemes and do not consider important protocol constraints of cluster-tree networks, for instance, buffer constraints and timing constraints of messages.

Finally, Koubaa et al. [13] proposed the Time Division Beacon Frame Scheduling (TDBS) approach, which defines a Superframe Duration Scheduling (SDS) algorithm for cluster-tree networks, considering a set of clusters with different superframe durations and beacon intervals. In this approach, clusters are organised within a defined major cycle, based on the Least Common Multiple (LCM) of the beacon periodicities for all clusters. The major cycle is divided in minor cycles, which are used to sequentially schedule all clusters. TDBS defines the start time for all clusters in a collision-free scheduling scheme. Also, it provides a set of rules to adequately assign duty-cycles for each cluster-head, based on the following constraints: (1) equal duty-cycles for leaf cluster-heads; (2) duty-cycles of parent cluster-heads must be greater or equal to the sum of duty-cycles of their child cluster-heads; and (3) the sum of duty-cycles must be smaller or equal to one. This scheme is adequate to schedule cluster-tree networks, where clusters have a similar number of child nodes and similar traffic load. Its main advantage is that it allows setting different beacon periodicities for the different sets of clusters.

Within this context, we can easily observe the shortage of mechanisms to properly allocate active communication periods within the CAP, in order to improve the throughput in cluster-tree networks avoiding common problems that can lead to undesirable network states (e.g., buffer overflows). This paper aims to provide a set of guidelines that enable network designers to efficiently define some of the most relevant operational parameters and message flow configurations for wide-scale cluster-tree networks.

## 3. System Model

In this work, we assume a set of $N_{nodes}$ sensor nodes organised according to a cluster-tree topology and randomly deployed along a wide-scale environment. Moreover, we consider that the network formation procedure ensures that all monitored environment is covered (no orphan nodes). The PAN coordinator (root of the tree and sink node) is a special FFD with a powerful central processing unit and an unlimited power source. It is responsible to trigger the network formation and acts as cluster-head for the first cluster, according to the IEEE 802.15.4 standard [41] and the ZigBee [6] specification.

Moreover, the cluster-tree network is composed of $N_{CH}$ coordinator nodes, acting as cluster-heads (CHs) of their clusters and periodically sending beacon frames to synchronise their child nodes. Each CH (except the root) belongs to two clusters, once as a child and once as a parent (i.e., a cluster-head).

**Figure 2:**
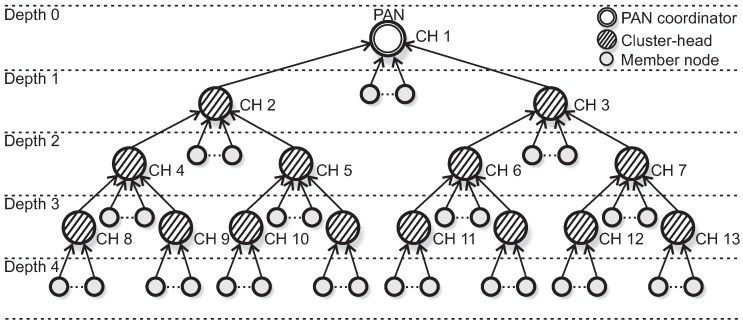
IEEE 802.15.4/ZigBee cluster-tree network.

We assume a set of static clusters, where there are no mobile nodes. Figure 2 illustrates an IEEE 802.15.4 cluster-tree wireless sensor network deployed in a specific wide-scale environment.

Within this context, we consider that cluster-heads share the same beacon interval, but a specific superframe duration is defined for each one of them. The evaluation of the superframe durations is performed through an offline scheduling approach, setted up during the network formation procedure. Therefore, the set of cluster-heads is characterized by:

(1) $$CH_{j}=\left(SD_{j},BI\right),\;\mathrm{for}\;1\leq j\leq N_{CH},$$

where $SD_{j}$ is the Superframe Duration of the cluster-head $CH_{j}$ and BI is the Beacon Interval for all clusters.

To avoid inter-cluster collisions caused by overlapping clusters, we assume that all active periods have been previously scheduled according to a time division beacon scheduling approach. For this, two different beacon scheduling schemes may be adopted:

- Top-down beacon scheduling, where superframe durations are ordered in a top-down direction. Firstly, the PAN coordinator, then clusters of the depth 1, and later clusters of the following depths are sequentially scheduled. This scheduling scheme prioritises downstream traffic.
- Bottom-up beacon scheduling: superframe durations are ordered in a bottom-up direction. Firstly, the deepest clusters (max depth of the tree) are scheduled, then clusters of next lower depth, following depth-by-depth until reaching the PAN coordinator. This scheduling scheme prioritises upstream traffic.

**Figure 3:**
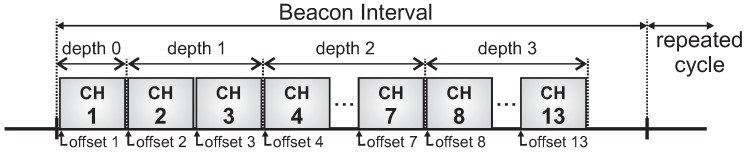
Top-down cluster active period scheduling.

**Figure 4:**
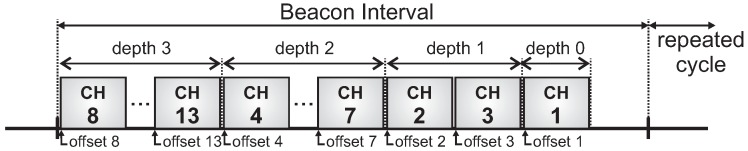
Bottom-up cluster active period scheduling.

Figure 3 illustrates a top-down scheduling, while Figure 4 illustrates a bottom-up scheduling for the network presented in Figure 2. Note that for the latter, clusters are scheduled using the reverse order of the top-down scheme.

For the sake of simplicity, no data aggregation or data fusion operations are performed by the cluster-heads. We also assume that the active portions of the clusters are composed only of contention access periods. Finally, we consider that whenever there are error sources affecting the wireless communication, these sources are statistically distributed along the communication environment. As the purpose of using a balanced bandwidth allocation scheme is to guarantee a fair distribution of the available communication resources (bandwidth), we do not consider the error behaviour of the communication channel as we consider that error sources equally affect all the network clusters.

For the message traffic model, we assume that sensing nodes periodically send messages to a *sink* node (PAN Coordinator) through the tree path routing (upstream traffic). Within this context, messages are modelled by a set S of *M* message streams:

(2) $$S=S_{1},S_{2},...,S_{M}$$

Messages generated at each message stream $S_{i}$ may be the consequence of periodic measurements of environment variables, being a message stream $S_{i}$ characterized as follows:

(3) $$S_{i}=\left(C_{i},P_{i}\right)$$

- where $P_{i}$ is the period of message stream $S_{i}$, which is not synchronised with the beacon arrivals at the sensor node;
- $C_{i}$ is the maximum length of each message in a message stream $S_{i}$. This parameter corresponds to the amount of time required to access the wireless channel, to transmit entirely the message, and to receive the acknowledgement, when required;
- the *k*-th message of a message streams $S_{i}$ is represented by $M_{i}^{k}$.

As messages are forwarded through the cluster-tree, the utilisation factor imposed by a specific message stream depends on the depth of its generator node. We define *U* as the total effective utilisation factor imposed by set S:

(4) $$U=\sum_{i=1}^{M}\frac{C_{i}}{P_{i}}\times depth_{i},$$

where $depth_{i}$ corresponds to the depth of the node that generates $S_{i}$ (considering the root node to be of depth 0, according to Figure 2).

## 4. Protocol Constraints

The allocation of superframe durations to each of the clusters is done according to a set of boundary equations, hereafter referred as protocol and buffer constraints. In this section, we model such a protocol and buffer constraints, considering the main constraints imposed by the IEEE 802.15.4 operation behaviour.

### 4.1. Length of the Beacon Interval

The length of the beacon interval is an important design parameter for setting-up a cluster-tree network. According to the IEEE 802.15.4 standard, the beacon interval and the superframe duration ($SD_{j}$) are defined as follows:

(5) $$\begin{array}{c} \left\{\begin{array}{c} BI=\alpha \times 2^{BO}\\ SD=\alpha \times 2^{SO},\;\mathrm{for}\;0\leq SO\leq BO\leq 14, \end{array}\right. \end{array}$$

where *α* corresponds to the *aBaseSuperframeDuration* MAC parameter. From Equation (5), it follows that:

(6) $$\alpha \times 2^{0}\leq SD\leq BI\leq \alpha \times 2^{14},$$

where BI and SD values are given by $2^{BO}$ and $2^{SO}$, respectively.

The beacon interval must be large enough to ensure that all desired superframe durations can be scheduled, but should also be as small as possible to reduce the end-to-end communication delay of message transfers:

(7) $$BI\geq \sum_{j=1}^{N_{CH}}SD_{j}$$

Considering that a message stream $S_{i}$ generates a new message to be transferred every period $P_{i}$, and that a node is able to send messages only during its cluster active period, there is a direct restriction imposed by the beacon interval upon the message stream periodicity.

**Figure 5:**
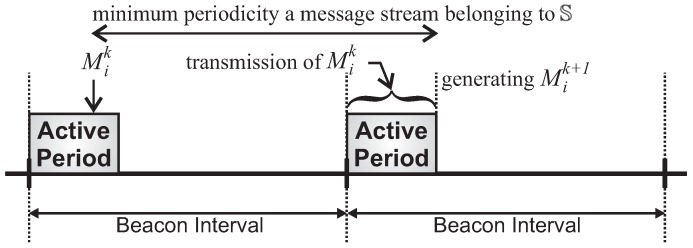
Length of beacon interval regarding message periodicities.

As the message generation period is not synchronised with the beacon arrivals, it may occur a message $M_{i}^{k}$ being generated immediately before the end of the active period of the cluster, and therefore there is no enough time to transmit that message (Figure 5). As a consequence, it would only be transmitted during the next active period.

Thus, considering *δ* as the maximum required time (contention time plus transmission and acknowledgement times) to transmit message $M_{i}^{k}$, to guarantee that $M_{i}^{k}$ may be transferred before the next message generation, period $P_{i}$ must be larger than the beacon interval plus *δ*. Considering set $P\;=\;\{P_{1},P_{2},...,P_{M}\}$ as the message periods of S, the following constraint applies upon the beacon interval:

(8) BI≤min{P}−δ,

where min{P} corresponds to the shortest period for the set S of defined message streams.

Considering that a cluster-tree network is multihop, a message to be transferred from source to sink must go through a sequence of clusters, during a sequence of scheduled active periods. In short, Figure 5 must consider both scheduling schemes illustrated in Figure 3 and Figure 4. A consequence of this multihop operating behaviour is that different scheduling approaches may or may not prioritise upstream traffic. The constraint defined in Equation (8) is adequate for a bottom-up scheduling scheme, as a message generated at the deepest source node is able to reach the sink node in just one beacon interval. However, using a top-down scheduling scheme, the generated message would take several beacon intervals to be delivered to the sink node. Thus, a protocol constraint that must be satisfied by the cluster-tree network would be as follows:

(9) $$depth_{MAX}\times BI\leq min\left\{P\right\}-\delta ,\;\mathrm{for}\;\text{top-down}\;\mathrm{scheduling}$$

Therefore, by combining Equations (7)–(9), a Protocol Constraint that must be satisfied by the superframe duration of any cluster in a cluster-tree WSN is:

(10) $$\begin{array}{c} \sum_{j=1}^{N_{CH}}SD_{j}\leq BI\leq \left\{\begin{array}{cc} P_{min}-\delta , & \mathrm{for}\;\text{bottom-up}\;\mathrm{scheduling}\\ \frac{P_{min}-\delta }{depth_{MAX}}, & \mathrm{for}\;\text{top-down}\;\mathrm{scheduling} \end{array}\right. \end{array}$$

**Figure 6:**
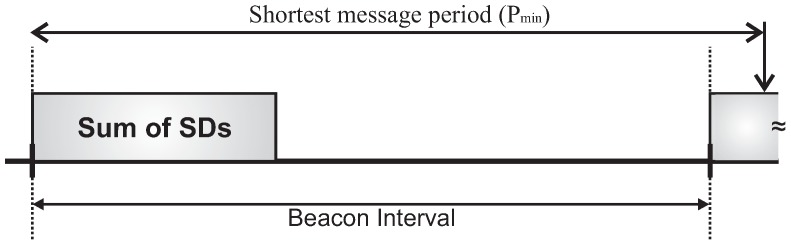
Longest Beacon interval.

**Figure 7:**
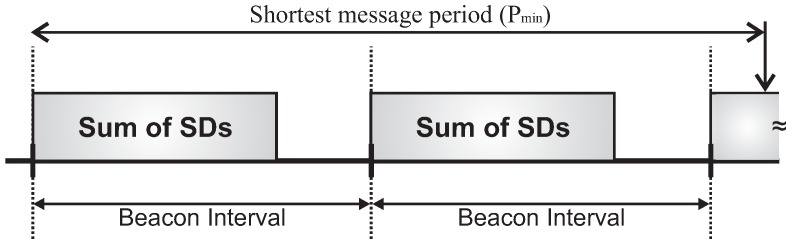
Shortest Beacon interval.

Considering both the protocol constraint (Equation (10)) and the way how MAC parameters can be setup (Equation (6)), the beacon interval may assume just an integer set of values ranging from the sum of superframe durations up to the shortest period of the set of message streams ($P_{min}$). This consideration represents an important design constraint for cluster-tree networks. Figure 6 and Figure 7 illustrate this design constraint.

On the one hand, the beacon interval should be set to a value close to the shortest message period (longest beacon interval), enhancing the network lifetime due to the reduction of energy consumption [42]. Considering this configuration mode, messages streams will have longer end-to-end communication delays (Figure 6).

On the other hand, the beacon interval could be set to a value close to the sum of superframe durations, considering the shortest beacon interval that still satisfies the protocol constraint (Figure 7). As a consequence, message streams would have shorter end-to-end communication delays, but with higher energy consumption of nodes.

### 4.2. Dimensioning Buffer Sizes

The adequate dimensioning of MAC buffers for the cluster-head nodes is a critical issue, because it has a clear impact upon message discards in cluster-tree networks [27]. Thus, it is important to define a boundary equation for the buffer usage, that should be imposed to the overall scheduling of the network. This boundary equation is relevant for avoiding message discards due to buffer overflows.

Regardless of any other issue, each cluster-head must be able to store, in the worst case, all messages generated by its descendant nodes (child nodes). That is:

(11) $$\eta \leq \sum_{i\in S_{below}^{j}}S_{i}\leq B_{j},$$

where $B_{j}$ is the size of the MAC buffer of cluster-head *j* (expressed in terms of number of messages), and *η* is the number of messages generated by the $S_{below}^{j}$ message streams located in the descendant nodes (child nodes) of $CH_{j}$ during one beacon interval.

Therefore, we have the following Buffer Constraint that must also be satisfied by a cluster-tree WSN:

(12) $$B_{j}\geq \sum_{n\in S_{below}^{j}}S_{n},\;\mathrm{for}\;1\leq n\leq N_{nodes}$$

We assume that messages in cluster-head buffers are priority ordered using a Rate-Monotonic priority scheme [43], i.e., message streams with shorter periods will have higher priorities.

## 5. Modelling the Transmission Time

As previously highlighted, the network load imposed by a message stream $S_{i}$ is constrained by the CSMA-CA parameters, such as channel access time and transmission time of a data frame. Due to the probabilistic behaviour of the CSMA-CA protocol, we consider the use of a set of communication models proposed by Kohvakka et al. [34] to estimate the frame transmission capacity within a superframe duration.

**Figure 8:**
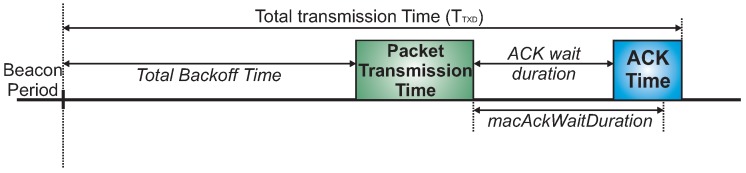
Transmission time duration of a data frame.

Within the active duration of a superframe, the transmission time ($T_{TXD}$) for a single data frame can be modelled as follows:

(13) $$T_{TXD}=T_{BOT}+T_{PACKET}+T_{radio\_transition}+T_{ACK}$$

where $T_{BOT}$ is the total backoff time, $T_{PACKET}$ is the packet transmission time, that is given by $\frac{L_{PACKET}}{D}$ (where $L_{PACKET}$ corresponds to packet frame length and *D* is the radio data rate). $T_{radio\_transition}$ corresponds to time duration that the radio takes to switch between different operating modes, for example, from sleep to receive mode and from receive to transmit mode. $T_{ACK}$ corresponds to the acknowledgement transmission time, that is given by $\frac{L_{ACK}}{D}$ (where $L_{ACK}$ corresponds to the ack frame length). Figure 8 illustrates the basic scheme for transmission of a single data frame. Note that the backoff period is aligned with the beacon interval and nodes must perform the backoff algorithm to transmit a data frame.

According to the CSMA-CA algorithm, a node needs to listen two CCAs before transmitting a packet (*CWinit* parameter default is 2). For this reason, Kohvakka et al. [34] model the probability ($P_{c}$) of a node to perform two consecutive CCAs as follows:

(14) $$P_{c}={\left(1-q\right)}^{2\times (N_{nodes}-1)},$$

where *q* corresponds to the probability that a node transmits a single message (with its ACK) during the CAP, which can be modelled as [34]:

(15) $$q=\frac{L_{PACKET}}{SD\times R}$$

The CSMA-CA algorithm also defines a maximum number of backoffs (*b*), which correspond to the number of attempts that the backoff algorithm is repeated in case of unsuccessfully CCA evaluations. This value is defined by *macMaxBackoffPeriod* MAC parameter and its default value is 4. Hence, the probability ($P_{s}$) of a node to perform a CCA with the maximum backoff number (*b*) can be modelled as:

(16) $$P_{s}=\sum_{NB=1}^{b}P_{c}\times {\left(1-P_{c}\right)}^{NB-1}$$

Then, the average backoff number (*r*) for each message can be modelled as [34]:

(17) $$r=\left(1-P_{s}\right)\times b+\sum_{NB=1}^{b}NB\times P_{c}\times {\left(1-P_{c}\right)}^{NB-1}$$

Also, the average backoff time ($T_{BO}$) for each message can be modelled as function of BE [34]:

(18) $$T_{BO}\left(BE\right)=\frac{2^{BE}-1}{2}\times T_{BOL},$$

where $T_{BOL}$ corresponds to the backoff period length and its value is defined by *aUnitBackoffPeriod* MAC parameter (default value is 20 *symbols*).

Therefore, the total backoff time can be modelled based on Equations (17) and (18) and considering that, for each backoff period, in average $\frac{3}{2}$ CCA assessments are performed [34]. Thus, the total backoff time ($T_{BOT}$) is given by:

(19) $$T_{BOT}=\frac{3}{2}r\left(T_{CCA}\right)+\sum_{NB=0}^{r-1}T_{BO}\left(min\left(macMinBE+NB,macMaxBE\right)\right),$$

where $T_{CCA}$ corresponds to the CCA listening time.

Within the context of this work, this set of analytical models is used to predict the number of messages transferred during a minimum superframe duration.

## 6. Timing Constraints

In this section, we define a set of timing boundary equations associated with the message response time, considering the supported set of message streams and the probabilistic behaviour of the CSMA-CA algorithm. Afterwards, these boundary equations are compared with the deadline of each message stream, in order to assess its schedulability. The main target of this set of boundary equations is to enable network designers to adjust the periodic message stream set and/or the protocol parameters of the cluster-tree network. As the proposed allocation schemes are intended to be used in probabilistic medium access networks, it is worth mentioning that target applications must be loss tolerant. Therefore, this response time analysis provides a probabilistic methodology to reduce the number of message drops due to buffer overflows and network traffic congestion.

The work presented in this paper considers earlier work from Lange et al. [22,23] and Agrawal et al. [44], which use similar approaches for the schedulability analysis of FlexRay and FDDI networks, respectively. The response time analysis calculations are based on earlier work presented by Audsley et al. [45], for the response time analysis of multi-task scheduling on mono-processors.

**Figure 9:**
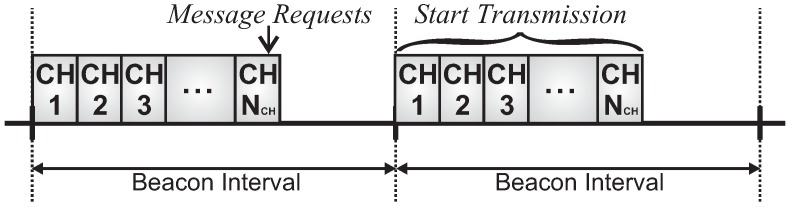
Worst-case scenario for cluster-tree networks.

For computing the boundary equation for the response time of a message stream, we consider a scenario where all messages streams are simultaneously generated just before the end of the cluster active period. In this case, all messages will be queued in the internal buffers of the nodes. Figure 9 illustrates this case, where the transmissions will only start in next active period scheduling, each one during its active period.

**Figure 10:**
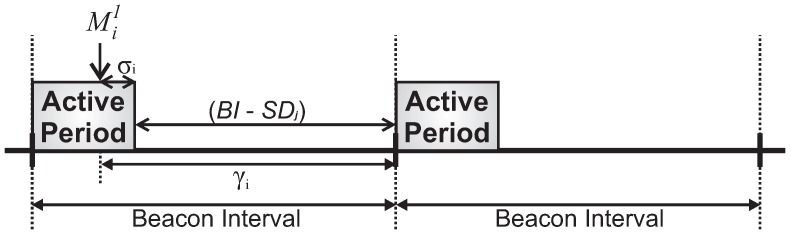
Local worst-case response time for a message stream.

In order to model a boundary equation for the response time ($R_{i}$) of a message stream $S_{i}$, we derive its probabilistic response time ($W_{i}$), which corresponds to the response time analysis for a specific cluster of any depth, assuming the probabilistic worst-case scenario for message $M_{i}^{k}$ of message stream $S_{i}$, according to Figure 10.

Note that, in a best-case scenario, this message will be transmitted to its parent cluster-head during the next cluster active period. This way, there is an initial delay $\gamma_{i}$, during which the message must wait for the next active period of its cluster. This initial delay can be expressed as follows:

(20) $$\gamma_{i}=\sigma_{i}+\left(BI-SD_{j}\right),$$

where $\sigma_{i}$ corresponds to a time interval immediately smaller than the transmission time for one message $M_{i}^{k}$ within the CAP and ($BI-SD_{j}$) corresponds to the inactive period of that cluster.

This message can suffer an interference ($\Theta_{i}$) from the higher priority message streams located in the descendant nodes of cluster-head $CH_{j}$ of the current active cluster *j*. In the best-case scenario, this interference corresponds to the sum of the transmission times of the set of higher priority message streams. However, if this sum exceeds the superframe duration, the related interference from the subsequent superframe must also be considered. Thus, we define ($\Theta_{i}$) for message $M_{i}^{k}$ as:

(21) $$\begin{array}{cc} \Theta_{i}^{1}\left(CH_{j}\right)={T_{TXD}}_{i} & +\left\lfloor \frac{\sum_{\begin{array}{c} h\in S_{below}^{j}\\ h\in hp(i) \end{array}}{T_{TXD}}_{h}}{SD_{j}}\right\rfloor \times \left(BI-SD_{j}\right)+\sum_{\begin{array}{c} h\in S_{below}^{j}\\ h\in hp(i) \end{array}}{T_{TXD}}_{h} \end{array}$$

(22) $$\begin{array}{c} \Theta_{i}^{w}\left(CH_{j}\right)={T_{TXD}}_{i}+\left\lfloor \frac{\sum_{\begin{array}{c} h\in S_{below}^{j}\\ h\in hp(i) \end{array}}\left\lceil \frac{\Theta_{i}^{w-1}\left(j\right)}{P_{h}}\right\rceil \times {T_{TXD}}_{h}}{SD_{j}}\right\rfloor \times \left(BI-SD_{j}\right)...\\ \;\;\;\;\;\;\;\;\;\;\;\;\;\;\;\;\;\;\;\;\;\;\;...+\sum_{\begin{array}{c} h\in S_{below}^{j}\\ h\in hp(i) \end{array}}\left\lceil \frac{\Theta_{i}^{w-1}\left(j\right)}{P_{h}}\right\rceil \times {T_{TXD}}_{h},\;\mathrm{for}\;w>1 \end{array}$$

Note that interference $\Theta_{i}$ is modelled as function of the active period of cluster-head $CH_{j}$, because message $M_{i}^{k}$ suffers interferences of multiple message streams $S_{below}^{j}$ every time it is forwarded along the cluster-tree path. The *w* iterations are performed until $\Theta_{i}^{w}=\Theta_{i}^{w-1}$.

Based on the interference time imposed by the highest priority message load, we derive the local worst-case response time ($W_{i}$) for message $M_{i}^{k}$ as:

(23) $$W_{i}=\gamma_{i}+\Theta_{i}^{w}$$

In addition, the message must traverse all the cluster-tree path from the source cluster until the PAN coordinator. Thus, the transmission delay towards the sink node takes into account the depth of the node that generated the message. Then, the total probabilistic worst-case response time ($R_{i}$) for message $M_{i}^{k}$ as function of the network depth (regarding the PAN coordinator) of the generation node of this message stream is derived as:

(24) $$R_{i}=\gamma_{i}+\sum_{d=depth_{i}-1}^{0}\Theta_{i}^{w}\left(CH_{j}\left(d\right)\right),$$

where CH(d) corresponds to cluster-head $CH_{j}$ of depth *d* that is responsible for forwarding message $M_{i}^{k}$ along the cluster-tree path.

Moreover, the response time analysis is also dependent on the active period scheduling scheme. For this reason, it is added an additional delay that is dependent on the used scheduling scheme. For the bottom-up scheduling scheme, where the upstream traffic is prioritised, this worst-case delay corresponds to the sum of active periods of all clusters. For the top-down scheduling scheme, where the message needs to use multiple beacon intervals before reaching the PAN coordinator, we consider a pessimistic worst-case delay that corresponds to the difference between one beacon interval and the superframe duration of the responsible cluster for forwarding the message at each depth of the cluster-tree network until reaching the PAN coordinator. These assumptions are appropriate, because they encompass worst-case scenarios for the scheduling schemes. Therefore, the probabilistic worst-case response time ($R_{i}$) can be derived as follows:

(25) $$\begin{array}{c} R_{i}=\left\{\begin{array}{cc} \gamma_{i}+\sum_{d=depth_{i}-1}^{0}\Theta_{i}^{w}\left(CH_{j}\left(d\right)\right)+\sum_{j=1}^{N_{CH}}SD_{j}, & \text{bottom-up}\;\mathrm{scheme}\\ \gamma_{i}+\sum_{d=depth_{i}-1}^{0}\Theta_{i}^{w}\left(CH_{j}\left(d\right)\right)+\sum_{d=depth_{i}-1}^{0}\left(BI-SD_{CH_{j}\left(d\right)}\right), & \text{top-down}\;\mathrm{scheme} \end{array}\right. \end{array}$$

Therefore, a boundary equation (timing constraint) that must be satisfied by the cluster-tree network scheduling can be defined as follows:

(26) $$R_{i}\leq P_{i},\;\mathrm{for}\;\forall S_{i}\in S$$

## 7. Superframe Duration Allocation Schemes

Finally, we derive a set of Superframe Duration Allocation (SDA) schemes, whose target is to improve the throughput of convergecast traffic in cluster-tree networks. The underlying reasoning is to estimate adequate values for the superframe durations and buffer sizes of each cluster coordinator, considering both the network requirements and the protocol and timing constraints. These allocation schemes can help system designers in what concerns the definition of network parameters, configuration of message streams and the need of using techniques such as data fusion or aggregation [46].

Two proportional allocation schemes for setting-up the superframe durations are proposed: (1) Load-SDA, based on the traffic load imposed by the cluster descendant nodes; and (2) Nodes-SDA, based on the number of descendant nodes. Load-SDA scheme is suitable for cluster-tree networks, where both the topology and the data traffic behaviour are known, whereas Nodes-SDA scheme is suitable for cluster-tree networks where only the topology is known.

The use of proportional allocation schemes to define the superframe durations ensures that adequate network resources (bandwidth) will be allocated to each cluster-head. Therefore, the use of such allocation schemes may avoid the network congestion and message discard issues that usually occur near the PAN coordinator [27]. Moreover, by defining adequate bandwidth and buffer sizes for cluster coordinators, the proposed allocation schemes may also guarantee a minimum level of QoS for message streams and a smaller energy consumption level for each of the network cluster-heads.

### 7.1. Proportional Allocation Scheme Based on the Message Load

In this subsection, we define a *Proportional Superframe Duration Allocation* scheme, which allocates bandwidth to a specific cluster based on its message load (Load-SDA). The reasoning is to proportionally allocate superframe durations to the cluster-heads based on the message traffic of their child nodes, including the accumulated message traffic of child coordinators. This scheme considers that both the network topology and the data traffic behaviour are known at system setup time. The Load-SDA scheme is described in Algorithm 1.

In the first step, the Load-SDA algorithm defines a value for the BI considering the constraint imposed by Equation (10). We model the number *X* of messages transferred during the minimum superframe duration $SD_{min}$ (step 2 in Algorithm 1) as follows:

(27) $$X=\left\lfloor \left(\frac{SD_{min}}{t_{TXD}}\right)\times {\left(1-P_{co}\right)}^{m}\right\rfloor ,$$

where $SD_{min}$ corresponds to the SO parameter equal to 0 (Equation (6)), $t_{TXD}$ corresponds to the total transmission time for a single message, and *m* represents the number of communicating nodes within a cluster multiplied by the probability *q* of a node to transmit a message at any time. For this analysis, we consider the number of communicating nodes within a cluster as the maximum number of nodes per cluster, which is a parameter defined before the formation phase of the cluster-tree network.

Thus, to define the $SD_{j}$ for cluster-head $CH_{j}$, the Load-SDA algorithm considers load *Y* imposed by all message streams hierarchically below the analysed cluster-head (step 3 in Algorithm 1, line 10), including its child nodes and the accumulated message load imposed by each child cluster-head, which is modelled as:

(28) $$Y=\sum_{i\in S_{below}}\frac{1}{\left\lfloor \frac{P_{i}}{BI}\right\rfloor },$$

where $\left\lfloor \frac{P_{i}}{BI}\right\rfloor$ corresponds to the maximum number of messages generated by $S_{i}$ during BI.
**Algorithm 1:** Load-SDA Algorithm
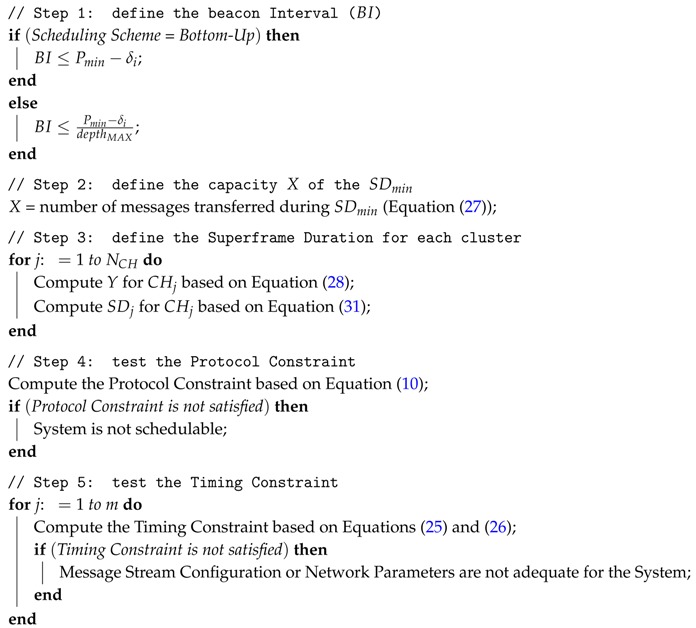



From Equations (27) and (28), the algorithm defines the necessary number of $SD_{min}$ required to handle the message load imposed by the supported message streams. Thus, we have:

(29) $$SD_{j}=\left\lceil \frac{Y}{X}\right\rceil \times SD_{min}$$

Following the constraints imposed for Equation (6), it follows that:

(30) $$SD_{j}=\alpha \times 2^{SO_{j}}$$

From Equations (29) and (30), to define the superframe duration ($SD_{j}$) for cluster-head $CH_{j}$ (step 3 in Algorithm 1, line 11), we derive the following equation to calculate its superframe order ($SO_{j}$):

(31) $$\begin{array}{c} \alpha \times 2^{SO_{j}}=\left\lceil \frac{Y}{X}\right\rceil \times \alpha \times 2^{0}\;\\ \;\;\;\;\;\;\;\;SO_{j}=\left\lceil \log_{2}\left(\left\lceil \frac{Y}{X}\right\rceil \right)\right\rceil \end{array}$$

After allocating a superframe duration for all cluster-heads, the protocol and timing constraints (Equations (10) and (26)) must be verified (steps 4 and 5 in Algorithm 1, lines 12–20). Case the protocol or the timing constraints are not satisfied, it means that the system may not be schedulable and it is necessary to modify the configuration of the network and/or the set of supported messages streams. Unfortunately, IEEE 802.15.4 standard provides a reduced flexibility to modify the values of the superframe duration and beacon Interval parameters. The main reason is that these parameters are described by SO and BO parameters, which are related to each other by a power of two. Thus, any adjustment of SO or BO parameters can significantly modify the values of SD and BI, respectively.

### 7.2. Proportional Allocation Scheme Based on the Number of Nodes

We also propose a *proportional Superframe Duration Allocation* scheme based on the number of descendant nodes (**Nodes-SDA**), which proportionally allocates a superframe duration for each cluster without considering the load imposed by each of its descendant nodes. Differently to the Load-SDA scheme, this allocation scheme is suitable for applications where the load imposed by each cluster is unknown. The Nodes-SDA scheme is described in Algorithm 2.
**Algorithm 2:** Nodes-SDA Algorithm
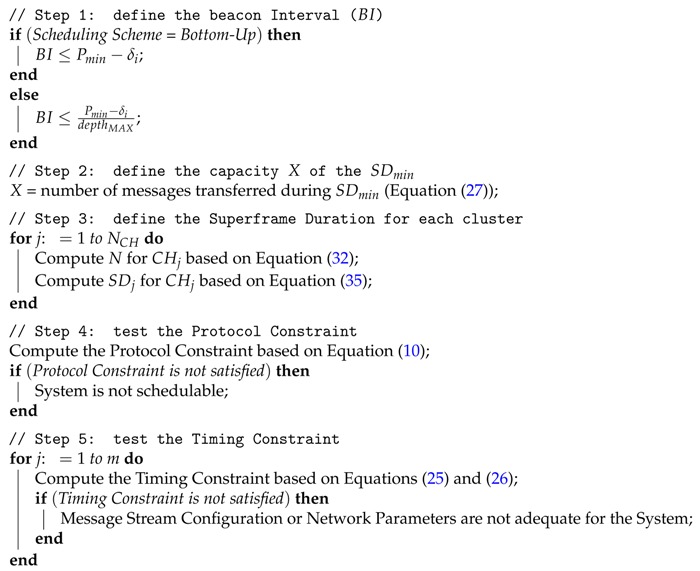



The first and second steps of the Nodes-SDA algorithm are similar to Load-SDA algorithm. The Beacon Interval and the number *X* of messages transferred during the minimum superframe duration $SD_{min}$ are defined according to Equations (10) and (27) (lines 1–7 in Algorithm 2). Thus, to define $SD_{j}$ for cluster-head $CH_{j}$, the Nodes-SDA algorithm just considers the number *N* of hierarchically descendant nodes below the analysed cluster-head (step 3 in Algorithm 1, line 10), including its child nodes and the accumulated child nodes of each child cluster-head:

(32) $$N=\sum_{S_{i}\in S_{below}}1,\;\mathrm{for}\;1\leq i\leq M$$

From Equations (27) and (32), the algorithm defines the necessary number of $SD_{min}$ to guarantee the message stream traffic. Thus, we have:

(33) $$SD_{j}=\left\lceil \frac{N}{X}\right\rceil \times SD_{min}$$

Following the constraints imposed by Equation (6), it follows that:

(34) $$SD_{j}=\alpha \times 2^{SO_{j}}$$

From Equations (33) and (34), to define the superframe duration ($SD_{j}$) for cluster-head $CH_{j}$ (step 3 in Algorithm 2, line 11), we derive the following equation to evaluate its respective superframe order ($SO_{j}$):

(35) $$\begin{array}{c} \alpha \times 2^{SO_{j}}=\left\lceil \frac{N}{X}\right\rceil \times \alpha \times 2^{0}\;\\ \;\;\;\;\;\;\;\;SO_{j}=\left\lceil \log_{2}\left(\left\lceil \frac{N}{X}\right\rceil \right)\right\rceil \end{array}$$

Finally, both the protocol and timing constraints (Equations (10) and (26)) must be verified (steps 4 and 5 in Algorithm 1, lines 12–20).

As Nodes-SDA scheme allocates superframe durations based just on the number of nodes, it may over-allocate durations for cluster-heads with lower message loads. This way, Nodes-SDA commonly allocated a larger sum of superframe durations, when compared to Load-SDA.

### 7.3. Illustrative Example

**Figure 11:**
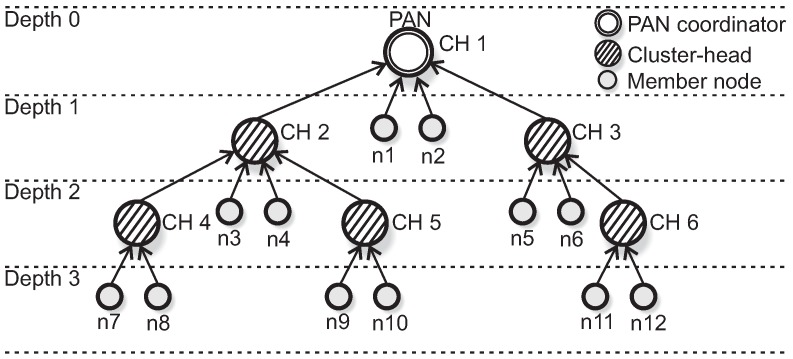
Cluster-tree network composed of 6 cluster-heads and 12 leaf nodes.

In this subsection, we present an example that illustrates the use of the Load-SDA allocation scheme. We consider a small example of a cluster-tree network composed of 6 cluster-heads, where each cluster is composed of 2 leaf nodes (Figure 11), and have a known traffic load. For the sake of simplicity, all values represented in this subsection are multiples of $SD_{min}$. We also assume that each leaf node handles one message stream; nodes with odd indexes handle message streams with periods equal to $60\times SD_{min}$ and nodes with even indexes generate message streams with periods equal to $70\times SD_{min}$. In this example, cluster-heads do not handle any message stream.

Based on the analysis presented in Section 5, we assume parameter *X* to be 2 messages per $SD_{min}$ (Equations (13) and (27)). For this example, we consider a bottom-up scheduling. Then, the Beacon Interval (BI) is set according to Equation (8). Considering that $Pi_{min}$ is $60\times SD_{min}$, BI is defined to be $32\times SD_{min}$ (BO = 5).

Thus, for cluster-head $CH_{6}$, the load imposed by its message streams (Equation (28)) is:

(36) $$Y=\sum_{i\in S_{below}}\frac{1}{\left\lfloor \frac{P_{i}}{BI}\right\rfloor }=\frac{1}{\left\lfloor \frac{60}{32}\right\rfloor }+\frac{1}{\left\lfloor \frac{70}{32}\right\rfloor }=1+\frac{1}{2}=\frac{3}{2}$$

Therefore, for $CH_{6}$, the superframe order $SO_{6}$ is:

(37) $$SO_{6}=\left\lceil \log_{2}\left(\left\lceil \frac{Y}{X}\right\rceil \right)\right\rceil =\left\lceil \log_{2}\left(\left\lceil \frac{3}{4}\right\rceil \right)\right\rceil =0$$

The superframe duration for $CH_{6}$ is $SD_{6}=\alpha \times 2^{0}$, that corresponds to $SD_{min}$. In fact, as cluster-head 6 has only two leaf nodes and $SD_{min}$ supports two messages, such a SD provides a reasonable bandwidth for this traffic. Following the same reasoning, superframe durations of cluster-heads 4 and 5 are also defined as $SD_{min}$.

For cluster-head $CH_{3}$, message load includes the load imposed by its child nodes (Equation (36)) and also by the child cluster-head $CH_{6}$ (accumulated load). The accumulated load is:

(38) $$Y=\sum_{i\in S_{below}}\frac{1}{\left\lfloor \frac{P_{i}}{BI}\right\rfloor }=2\times \frac{1}{\left\lfloor \frac{60}{32}\right\rfloor }+2\times \frac{1}{\left\lfloor \frac{70}{32}\right\rfloor }=2+1=3$$

Thus, for $CH_{3}$, the superframe order $SO_{3}$ is:

(39) $$SO_{3}=\left\lceil \log_{2}\left(\left\lceil \frac{Y}{X}\right\rceil \right)\right\rceil =\left\lceil \log_{2}\left(\left\lceil \frac{3}{2}\right\rceil \right)\right\rceil =1$$

The superframe duration for $CH_{3}$ is $SD_{3}=\alpha \times 2^{1}$, which corresponds to $2\times SD_{min}$. Table 1 shows the superframe duration for all clusters.

After defining the superframe duration for all cluster-heads, the protocol and timing constraints need to be verified. Based on Equation (10), the protocol constraint is respected, as follows:

(40) $$\begin{array}{cc} \sum_{j=1}^{N}SD_{j} & \leq BI\leq P_{min}-\delta_{i} \end{array}$$

(41) $$\begin{array}{cc} 17\times SD_{min}\leq 32\times & SD_{min}\leq 60\times SD_{min}-\frac{1}{2}\times SD_{min} \end{array}$$

(42) $$\begin{array}{cc} 17 & \leq 32\leq 60-\frac{1}{2} \end{array}$$

Regarding the timing constraint, as an example, we present the probabilistic response time analysis for message stream $S_{10}$ (depth 3). For this work, we assume that message streams with the same period of $S_{10}$ are considered for the set of the higher priority message streams. Thus, for depth 3 ($CH_{5}$), the set of higher priority message streams is composed only of message stream $S_{9}$. Thus, we have:

(43) $$\begin{array}{cc} \Theta_{10}^{1}\left(CH_{5}\right) & =\frac{1}{2}+\left\lfloor \frac{\frac{1}{2}}{1}\right\rfloor \times 31+\frac{1}{2}=1 \end{array}$$

(44) $$\begin{array}{cc} \Theta_{10}^{2}\left(CH_{5}\right) & =\frac{1}{2}+\left\lfloor \frac{\left\lceil \frac{1}{60}\right\rceil \times \frac{1}{2}}{1}\right\rfloor \times 31+\left\lceil \frac{1}{60}\right\rceil \times \frac{1}{2}=1 \end{array}$$

For depth 2 ($CH_{2}$), the set of higher priority message streams is composed of message streams $S_{3}$, $S_{4}$, $S_{7}$, $S_{8}$ and $S_{9}$. Thus, we have:

(45) $$\begin{array}{cc} \Theta_{10}^{1}\left(CH_{2}\right) & =\frac{1}{2}+\left\lfloor \frac{\frac{1}{2}\times 5}{4}\right\rfloor \times 28+\frac{1}{2}\times 5=3 \end{array}$$

(46) $$\begin{array}{cc} \Theta_{10}^{2}\left(CH_{2}\right) & =\frac{1}{2}+\left\lfloor \frac{\left\lceil \frac{3}{60}\right\rceil \times \frac{1}{2}\times 3+\left\lceil \frac{2}{70}\right\rceil \times \frac{1}{2}\times 2}{4}\right\rfloor \times 28+\left\lceil \frac{3}{60}\right\rceil \times \frac{1}{2}\times 3+\left\lceil \frac{3}{70}\right\rceil \times \frac{1}{2}\times 2=3 \end{array}$$

In turn, for depth 1 ($CH_{1}$), the set of higher priority message streams is composed of message streams $S_{1}$, $S_{2}$, $S_{3}$, $S_{4}$, $S_{5}$, $S_{6}$, $S_{7}$, $S_{8}$, $S_{9}$, $S_{11}$ and $S_{12}$. Thus, we have:

(47) $$\begin{array}{cc} \Theta_{10}^{1}\left(CH_{1}\right) & =\frac{1}{2}+\left\lfloor \frac{\frac{1}{2}\times 11}{8}\right\rfloor \times 24+\frac{1}{2}\times 11=6 \end{array}$$

(48) $$\begin{array}{cc} \Theta_{10}^{2}\left(CH_{1}\right) & =\frac{1}{2}+\left\lfloor \frac{\left\lceil \frac{6}{60}\right\rceil \times \frac{1}{2}\times 6+\left\lceil \frac{6}{70}\right\rceil \times \frac{1}{2}\times 5}{8}\right\rfloor \times 24+\left\lceil \frac{6}{60}\right\rceil \times \frac{1}{2}\times 6+\left\lceil \frac{6}{70}\right\rceil \times \frac{1}{2}\times 5=6 \end{array}$$

Finally, applying Equation (25) for the case of bottom-up scheduling, we have:

(49) $$\begin{array}{c} R_{i}=\sum_{j=1}^{N_{CH}}SD_{j}+\gamma_{i}+\sum_{d=1}^{depth_{i}}\Theta_{i}^{w}\left(CH_{j}\left(d\right)\right) \end{array}$$

(50) $$\begin{array}{c} R_{10}=\sum_{j=1}^{N_{CH}}SD_{j}+\sigma_{10}+\left(BI-SD_{5}\right)+\Theta_{10}^{w}\left(CH_{5}\right)+\Theta_{10}^{w}\left(CH_{2}\right)+\Theta_{10}^{w}\left(CH_{1}\right) \end{array}$$

(51) $$\begin{array}{c} R_{10}=17+\frac{1}{2}+\left(32-1\right)+1+3+6=58,5 \end{array}$$

considering $\sigma_{10}$ as approximately $\frac{1}{2}\times SD_{min}$. Thus, the timing constraint for message stream $S_{10}$ is satisfied, as follows:

(52) $$\begin{array}{cc} R_{10} & \leq P_{10} \end{array}$$

(53) $$\begin{array}{cc} 58,5 & \leq 70 \end{array}$$

Table 2 shows the timing constraints for all message streams, where it is clear that all deadlines can be met (a probabilistic guarantee, as the underlying message duration is probabilistic).

The purpose of this example is just to illustrate the use of allocation scheme Load-SDA. Noticeably, for a real cluster-tree network, this analysis must be extended to the overall set of cluster-heads.

## 8. Simulation Assessment

Finally, this section presents a simulation assessment of the superframe duration allocation schemes proposed in this paper. The main objective is to analyse the network behaviour when applying the proposed allocation schemes and to compare it with the case of both a state-of-the-art allocation scheme (based on work done by [13]) and a similar-duration superframe duration allocation scheme.

For this simulation assessment, we have implemented the CT-SIM simulation model [47] for cluster-tree networks using the Castalia Simulator [48]. Castalia (The Castalia Simulator for Wireless Sensor Network: https://castalia.forge.nicta.com.au.) is an open-source discrete event simulator for WSNs, Body Area Networks (BAN) and general low-power embedded networks, that was developed at National ICT Australia (NICTA) and is based on the OMNeT++ platform. Castalia is a very popular simulator, widely used by researchers and developers to test their protocols using a realistic wireless channel and radio models [49]. Castalia implements an advanced wireless channel model, based on empirically measured data. Also, the simulator provides radio models based on real low-power communication radios. Moreover, important features to simulate WSNs are available, such as: realistic node behaviour, node *clock drift*, and energy consumption models.

Castalia provides an IEEE 802.15.4 model. However, this model is quite limited. Basically, it only implements the CSMA-CA functionality and a beacon-enabled star topology, including an association procedure, direct data transfer mode, and GTS communication. The CT-SIM simulation model [47] provides a set of models running upon Castalia, that includes a series of multi-hop functionalities, such as: cluster-tree formation procedure, network scheduling, hierarchical addressing scheme, direct and indirect data communication, collision domain definition, data communication to the sink node (PAN coordinator), and the proposed superframe duration allocation schemes.

### 8.1. Simulation Environment

**Figure 12:**
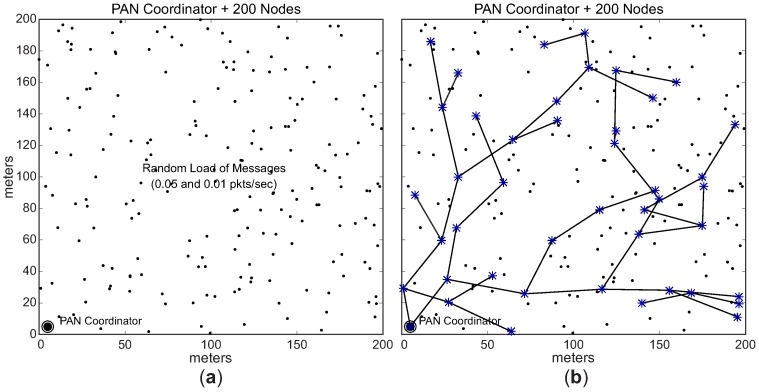
Configuration of the *unconditioned Scenario*: (**a**) Randomly distributed data rates; (**b**) Unconditioned cluster-tree network.

For this simulation assessment, it was considered a communication environment with a size of 200 m × 200 m, composed of 201 sensor nodes (one PAN coordinator, plus 200 sensing nodes). The PAN coordinator was located in position 5 m × 5 m of the environment, while 200 sensing nodes were randomly deployed. The PAN coordinator node was deployed in the corner of the environment, in order to build deep cluster-tree networks. Figure 12a illustrates an example of the simulation environment used in this assessment.

Regarding the monitoring traffic, sensing nodes generate periodic messages and send them to sink node (PAN coordinator). For the sake of simplification, we defined that each sensing node supports one message stream and that PAN coordinator does not generate any traffic itself. Each sensing node generates 1000 data messages, sending them to the PAN coordinator according to the rules defined by IEEE 802.15.4/ZigBee data communication. Thus, data messages are forwarded along the cluster-tree network according to the tree routing protocol. Importantly, cluster-heads do not perform any data aggregation or fusion mechanism, which implies that all monitoring traffic is forwarded towards the sink node. In order to generate different message loads for the cluster-heads, we defined two different data rates for the set of message streams: a higher data rate (0.05 pkts/s—periodicity of 20 s), and a lower data rate (0.01 pkts/s—periodicity of 100 s).

The cluster-tree formation process is based on the IEEE 802.15.4 standard/ZigBee specifications. The PAN coordinator (defined as depth 0 of the cluster-tree network) is responsible to trigger the formation procedure, by building its own cluster and acting as cluster-head. We defined the maximum number of child nodes per cluster to be 6 (six). For this simulation assessment, we have defined two cluster-tree formation procedures, in order to create two different simulation scenarios: an unconditioned cluster-tree (hereafter called *unconditioned Scenario*) and a conditioned cluster-tree (hereafter called *conditioned Scenario*).

In the first scenario (unconditioned formation), each CH (including the PAN coordinator) can select a maximum number of 2 (two) candidate child nodes to be cluster-heads. The selection of CH candidates is randomly performed and the cluster-tree network can grow in any direction. Each CH candidate can build its own cluster, following the same rules. The data rates are randomly distributed along the sensing nodes in the network environment. Figure 12 shows an example of the *unconditioned Scenario*. Figure 12a illustrates the data rates randomly distributed along the environment, while Figure 12b illustrates an example of the physical topology for the *unconditioned Scenario*.

In the second scenario (conditioned formation), we have equally divided the environment in two different load zones: high load zone, and low load zone. Nodes located in the high load zone are configured with data rate of 0.05 pkts/s (higher data rate), whereas nodes located in the low load zone are configured with data rate of 0.01 pkts/s (lower data rate).

**Figure 13:**
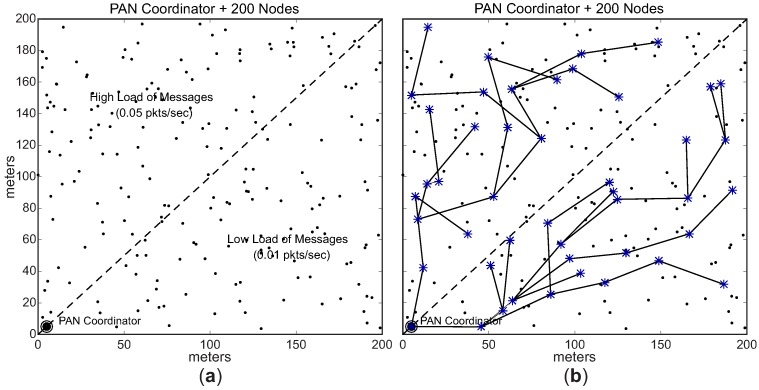
Configuration of the *conditioned Scenario*: (**a**) Data rates distributed in two load zones; (**b**) Conditioned cluster-tree network.

Considering these two different load zones, the cluster-tree formation process is started by the PAN coordinator, which selects one CH candidate in the high load zone and another candidate located in the low load zone. Following, each cluster-head can select a maximum number of 3 (three) CH candidates, that must also be located in the same load zone of their parent CHs. Therefore, we have a conditioned cluster-tree network, where one branch is built along the high load zone and the another branch is built along the low load zone. Figure 13 shows this *conditioned Scenario*. Figure 13a illustrates the two defined load zones (high and low load zones), while Figure 13b illustrates an example of a physical topology for the *conditioned Scenario*. Table 3 summarises the main features of the *unconditioned* and *conditioned Scenarios*.

For this simulation assessment, we used the ZigBee-based *hierarchical addressing* scheme, in which each CH has its own sequential address block. Regarding the active period scheduling, we used a typical time division scheme. For the sake of simplification, we have used a bottom-up scheduling scheme, which prioritises the monitoring traffic (from leaf cluster-heads toward the PAN coordinator). Basically, the main difference between bottom-up and top-down scheduling schemes is related to the the protocol constraint, as the top-down scheme imposes more demanding beacon interval restrictions.

Regarding the node’s features, we have adopted the CC2420 (Texas Instruments/Chipcon CC2420 Datasheet: http://www.ti.com/product/CC2420/technicaldocuments). radio model, which is compliant with the IEEE 802.15.4 standard. Furthermore, we adopted a linear energy model provided by Castalia and the initial energy for all nodes was set to 18.720 Joules (typical energy for two AA batteries). We also adopted the *unit disc* model as the radio propagation model, where the range of the disk was defined to be 55 m. For the interference model, we use a simple interference model provided by Castalia, where concurrent transmissions generate collisions at the receiver. Table 4 summarises the most important configuration parameters used in the simulations.

#### Performance Metrics

The aim of this simulation assessment is to evaluate the network behaviour according to well-defined metrics, both for the unconditioned and conditioned scenarios. In a first set of experiments, the behaviour of Load-SDA and Nodes-SDA allocation schemes are compared against both a state-of-the-art allocation scheme, and a standard allocation scheme. In a second set of experiments, the behaviour of the Load-SDA allocation scheme is compared just against the proposed Nodes-SDA allocation, in order to highlight the differences between these two schemes. The following acronyms were used to identify the allocation schemes.

- *Load-SDA*: Allocation scheme based on the message load (Load-SDA), where the X parameter value (Equation (27)) was considered to be 2 (two) messages, and the main CSMA-CA parameters were defined according to their default values ($SD_{min}$ corresponds to 15.36 ms, assuming a network with bit rate of 250 kbps, frequency band of 2.4 GHz, and one symbol as 4 bits).
- *Nodes-SDA*: Allocation scheme based on the number of nodes (Nodes-SDA). In this case, similar rules as defined for Load-SDA scheme were considered.
- *SOA-SDA*: State-of-the-art Allocation scheme based on the work proposed by [13], considering a similar beacon interval (BI) value for all the clusters. For this scheme, leaf cluster-heads were configured with the minimum SD (considering SO = 0) and all other cluster-heads were configured according to the rules defined in [13]. The BI value was defined according to the same constraints imposed by the Load-SDA allocation scheme, according to Equation (10).
- *STD-SDA*: Similar-Duration Allocation scheme. For this scheme, all clusters in the network share the same Superframe Duration and Beacon Interval. The BI value was defined according to Equation (10). The SD value was defined as the upper average of the SOs (SuperframeOrder), as defined by the *Load-SDA* scheme for all clusters (respecting the constraint defined by Equation (5)).

In order to analyse the network behaviour, the following performance metrics were used:

- *Message Discard Rate*: percentage of discarded messages due to buffer overflows in the cluster-heads, considering the number of discarded messages vs. the number of messages that arrived to the cluster-head.
- *Message Loss Rate*: percentage of messages lost during the communication, considering the number of messages successfully received at the destination node and the number of messages generated by the source node (encompasses both discarded messages and messages lost due to collisions).
- *Average End-to-end Delay*: time interval between data frame generation at the application layer of the source node and its reception at the application layer of sink node.
- *Energy Consumption*: average energy consumption of the overall network.

For each of the allocation schemes, 10 different simulations were performed for each scenario (unconditioned and conditioned) with different sets of random variables. Therefore, presented results correspond to the average results obtained from this set of simulations.

### 8.2. Results and Discussion

Firstly, we present some information about the cluster-tree network formation for each of the defined scenarios. Table 5 shows the average number of generated clusters during the cluster-tree formation, the average maximum depth of the cluster-tree network, and the average number of children per cluster.

The main target of the proposed proportional SDA schemes is to allocate adequate communication resources (superframe durations and buffer sizes) for the cluster-heads, in order to avoid network congestion and message discards due to buffer overflows. Therefore, the buffer occupancy is an important performance metric to evaluate the proposed allocation schemes. Table 6 shows the considered buffer sizes for the cluster-heads at each depth of the cluster-tree (average values for the different cluster-heads located at each level of the different tree branches).

Load-SDA and Nodes-SDA schemes define buffer sizes for cluster-heads that are proportional to the number of descendant nodes, according to the defined buffer constraint (Equation (12)). As STD-SDA and SOA-SDA allocation schemes do not provide any mechanism to define buffer sizes, we have set the length of internal buffers to a value equal to the total number of sensing nodes (200).

Table 7 illustrates the average SO parameter values for cluster-heads, considering their depths in the cluster-tree network. Note that, as both SOA-SDA, Load-SDA and Nodes-SDA schemes allocate superframe durations based on the imposed traffic, allocations are proportional to depth: clusters closer to the PAN coordinator have higher superframe durations. The difference between these three schemes is that in Load-SDA scheme, superframe durations are based on the traffic load of the cluster itself and of its descendant nodes, while Nodes-SDA scheme only considers the number of descendant nodes. Instead, SOA-SDA allocation scheme imposes that a parent cluster-head must have duty-cycle greater or equal to the sum of duty-cycles of its child cluster-heads, leading to slightly different values for the allocations. Finally, STD-SDA scheme allocates the same superframe duration for all cluster-heads.

**Figure 14:**
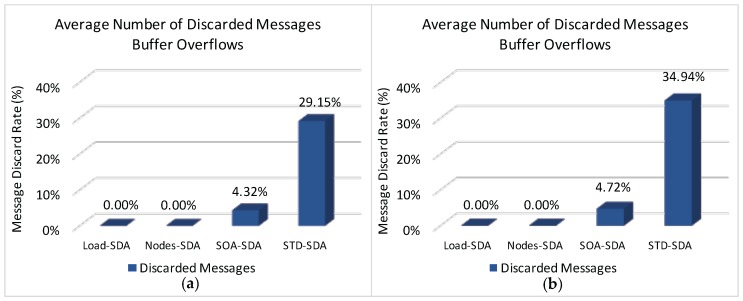
Average discard message rate due to buffer overflows: (**a**) Unconditioned Scenario; (**b**) Conditioned Scenario.

Figure 14 illustrates the average rate of discarded messages due to buffer overflows for the overall network, when applying the four allocation schemes to the defined scenarios.

It can be observed (Figure 14) that both Load-SDA and Nodes-SDA schemes behave adequately for both communication scenarios, considering the defined set of message streams. All CHs were able to forward their messages and no messages were discarded due to buffer overflows. This behaviour highlights one of the major advantages of the Load-SDA and Nodes-SDA allocation schemes, where both superframe durations and buffer sizes are dimensioned according to the network load and number of nodes of each of the branches of the cluster-tree network, respectively. On the other hand, the STD-SDA scheme discarded 30%–35% of the messages due to the allocation of inappropriate superframe durations and also due to the inability of the cluster-heads to temporarily store the accumulated messages in their internal buffers, despite the larger number of allocated buffer resources. The SOA-SDA scheme presents a much smaller number of discarded messages due to buffer overflows (4%–5% of messages), as it considers the adjustment of the superframe durations according to the depth of each cluster-head in the cluster-tree network. These results clearly highlight that an equal allocation of the superframe durations is not adequate for cluster-tree networks, as it does not consider the tree topology effects of the network.

**Figure 15:**
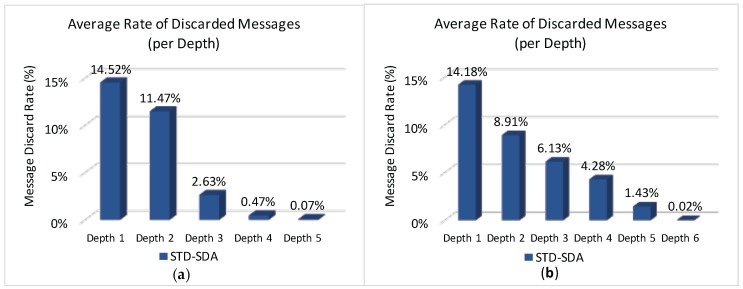
Average message discard rate (per depth) due to buffer overflows for the STD-SDA scheme: (**a**) Unconditioned Scenario; (**b**) Conditioned Scenario.

Considering that both STD-SDA allocation schemes have discarded messages, it is important to check where those messages were discarded. Figure 15 illustrates the average number of discarded messages for the STD-SDA allocation scheme, as function of the CHs’ depth. As expected, the number of discarded messages is higher for cluster-heads located at depth 1, followed by cluster-heads of depth 2, and so on. In fact, as convergecast traffic is forwarded through the cluster-tree towards the PAN coordinator, the trend is that cluster-heads near the PAN coordinator will be more congested, where the network performance will be substantially affected. This behaviour is observed for both unconditioned and conditioned scenarios.

**Figure 16:**
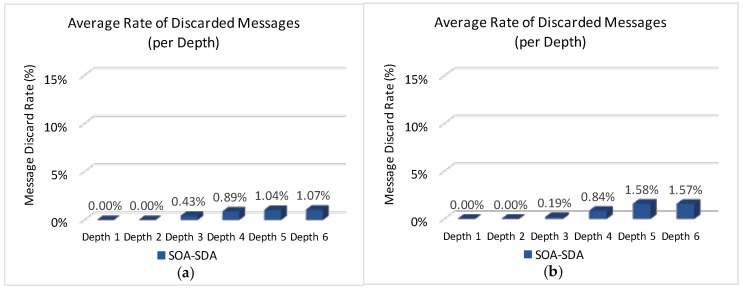
Average message discard rate (per depth) due to buffer overflows for the SOA-SDA scheme: (**a**) Unconditioned Scenario; (**b**) Conditioned Scenario.

Figure 16 illustrates the average number of discarded messages as function of the CHs’ depth for the SOA-SDA allocation scheme. It can be observed that, this scheme adequately allocated superframe duration values for the clusters located closer to PAN coordinator, but not for the deepest clusters. This problem could be solved by increasing superframe duration values for leaf cluster-heads. However, as the SOA-SDA allocation scheme defines that the duty-cycle of a parent cluster-head must be greater or equal to the sum of duty-cycles of child cluster-heads, increasing the allocation values for the leaf cluster-heads could lead to the non-fulfilment of the overall protocol constraint (Equation (10)).

**Figure 17:**
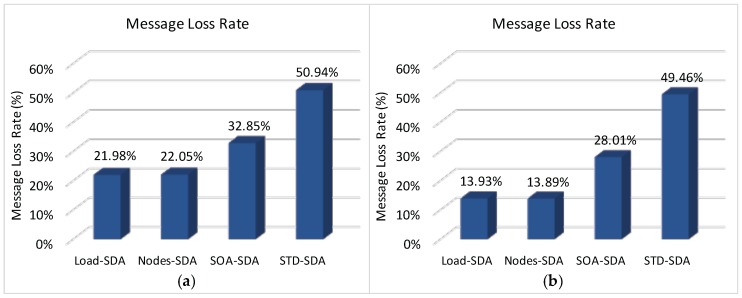
Message loss rate for the SDA schemes: (**a**) Unconditioned Scenario; (**b**) Conditioned Scenario.

Finally, Figure 17 illustrates the total message loss rate (considering both message collisions and message discards due to buffer overflows) for each of the defined allocation schemes. As it can be observed, the number of discarded messages due to buffer overflows strongly influences the number of lost messages, decreasing the number of successfully delivered messages. Comparing results of Figure 14 and Figure 17, it is clear that the message loss rate due to message collisions is around 22%–28% for the all allocation schemes. The main reason for this high number of message losses is due to the (default) CSMA/CA parameters used for the simulation assessment. As previously shown in [9,36], the default parameter values used for *macMinBE*, *macMaxBE* and *CW* (Contention Window) can easily lead to a high number of message collisions, for a number of sensor devices as low as 6 devices per cluster-head.

According to this simulation assessment, it becomes clear the importance of defining adequate active communication periods and buffer sizes for the cluster-heads. An adequate allocation of superframe durations for the different clusters can significantly improve the network behaviour.

We have also assessed the average end-to-end delay for convergecast traffic, in order to evaluate the influence of the buffer sizes and superframe allocation schemes. Considering that the beacon interval parameter can directly influence the behaviour of the network (refer to Section 4.1), we have assessed the different possibilities for the BI adjustment and its impact upon the network behaviour (end-to-end message delays and energy consumption of the nodes). As previously mentioned, the SOA-SDA allocation scheme was constrained to the use of a single beacon interval for all the clusters. Nevertheless, this would be the expected parameter settings for the case where all the supported traffic has the same periodicity.

**Figure 18:**
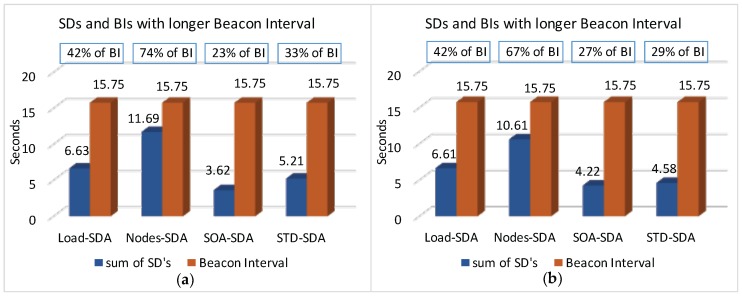
Beacon Interval and average sum of superframe durations for a network with a longer beacon interval in the: (**a**) Unconditioned Scenario; (**b**) Conditioned Scenario.

Firstly, we have considered the case where the beacon interval is set to a value close to the shorter message period (longer beacon interval). Figure 18 illustrates both the beacon interval and the average sum of superframe durations defined for each of the allocation schemes (absolute average values and the percentage of the sum of superframe durations regarding to BI).

**Figure 19:**
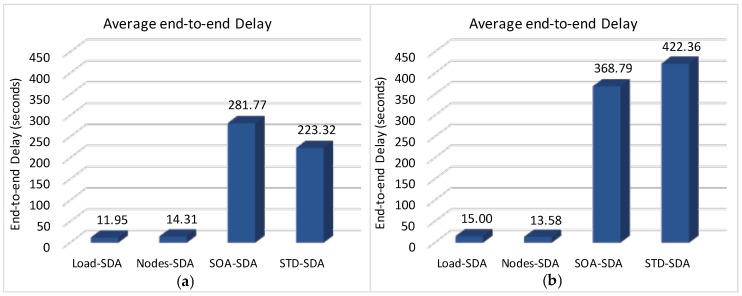
Average end-to-end communication delay for a network with a longer Beacon Interval in the: (**a**) Unconditioned Scenario; (**b**) Conditioned Scenario.

Figure 19 illustrates the average end-to-end communication delay for a network with longer Beacon Interval. It can be observed that end-to-end delays for the SOA-SDA and STD-SDA schemes are remarkably higher than for the case of the Load-SDA and Nodes-SDA schemes. The main reason is that, as the network is congested, messages are remaining more time in the internal buffers of cluster-heads. Therefore, message transfers require more beacon intervals to be forwarded, increasing their end-to-end communication delays. As the network, with the proposed Load-SDA and Nodes-SDA allocation schemes, does not face any congestion, messages can flow along the tree until the sink node, being their end-to-end communication delays slightly smaller than the beacon interval.

**Figure 20:**
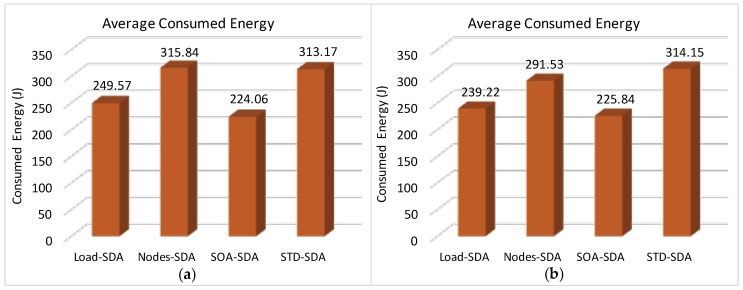
Average consumed energy for the allocation schemes: (**a**) Unconditioned Scenario; (**b**) Conditioned Scenario.

Figure 20 illustrates the average total energy consumption for a network with longer Beacon Interval. The energy consumption is mainly related to two factors: the time interval during which the nodes remain active, and the activities performed by them. As the active period (sum of superframe durations) of SOA-SDA is smaller than for Load-SDA scheme, it has a slightly better performance. Note also that, the average energy consumption for the Nodes-SDA scheme is larger than for both the Load-SDA and SOA-SDA schemes, considering both scenarios.

In what concerns the STD-SDA allocation scheme, it is important to highlight that the energy consumption is proportional to the number of non-discarded messages. Therefore, as there is a large number of messages being discarded due to buffer overflows, there is a consequent reduction of the energy consumption of the overall network.

**Figure 21:**
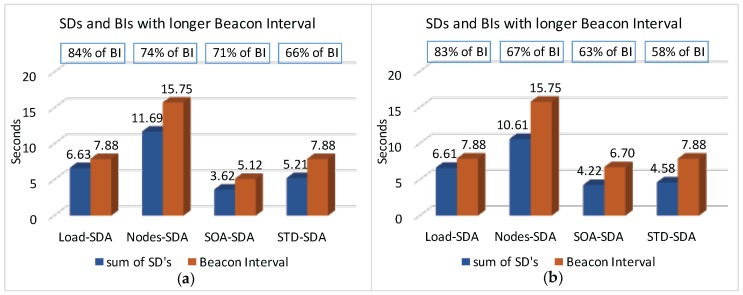
Average sum of superframe durations and beacon interval for the allocation schemes with adjustment of BI: (**a**) Unconditioned Scenario; (**b**) Conditioned Scenario.

**Figure 22:**
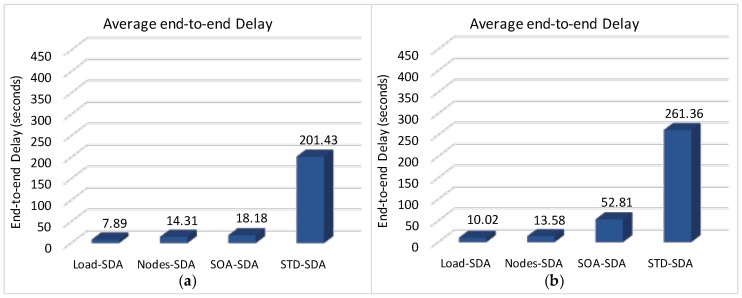
Average end-to-end delay for the monitoring traffic with a shorter beacon interval: (**a**) Unconditioned Scenario; (**b**) Conditioned Scenario.

Figure 21 illustrates the beacon intervals and the average sum of superframe durations defined for each of the allocation schemes, considering the shorter beacon interval (absolute average values and percentage of the sum of superframe durations regarding to BI). In this case, the adjustment of the beacon interval was performed for all allocation schemes (except for the Nodes-SDA scheme), being that the SOA-SDA scheme had the smaller average beacon interval. For the Nodes-SDA scheme, if the beacon interval (in terms of beacon order) is decreased, the protocol constraint would no longer be respected. Figure 22 shows the average end-to-end communication delay for a network with a shorter BI.

**Figure 23:**
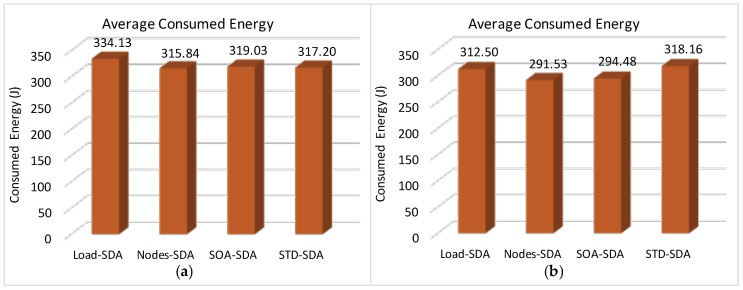
Average consumed energy for the allocation schemes with a shorter beacon interval: (**a**) Unconditioned Scenario; (**b**) Conditioned Scenario.

As it can be observed, the average end-to-end communication delay is significantly smaller when considering the reduction of the beacon interval, according to the reasoning previously presented in Section 4.1. In fact, a shorter beacon interval always corresponds to shorter end-to-end communication delays. It is clear that there is a significant improvement of the end-to-end communication delay for the SOA-SDA allocation scheme, compared to the case with longer beacon interval. This behaviour is due to the fact that considering a shorter beacon interval, messages can be dispatched in a shorter time interval, decreasing network congestion and message discards. However, the energy consumption will increase, due to the higher activation rate of the sensor nodes. Figure 23 illustrates the average total energy consumption for the allocation schemes, considering both unconditioned and conditioned allocation schemes with a shorter beacon interval. It can be concluded that, for the case of shorter beacon intervals, the Load-SDA, Nodes-SDA and SOA-SDA schemes have a better performance when compared to the STD-SDA schemes (similar energy consumption, but significantly smaller end-to-end communication delays).

**Figure 24:**
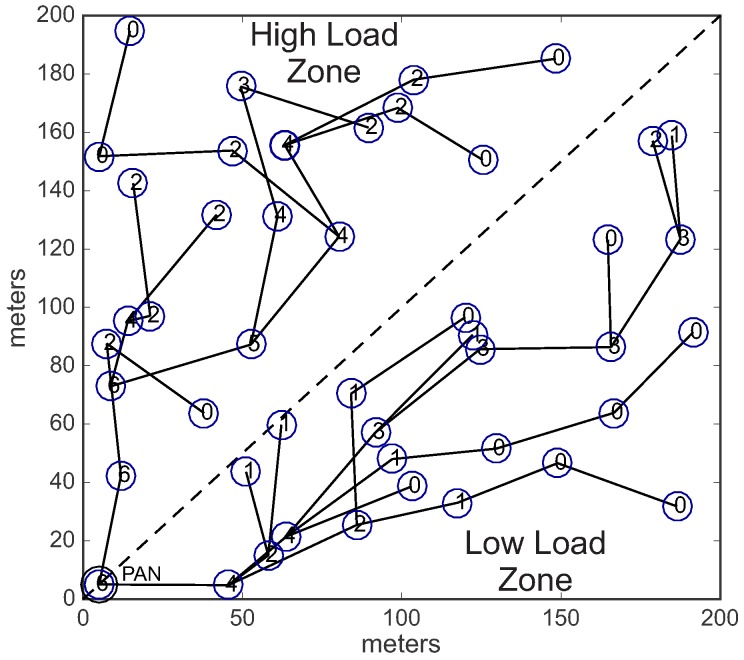
An example of Superframe Order configuration for all cluster-heads, using Load-SDA scheme (conditioned Scenario).

**Figure 25:**
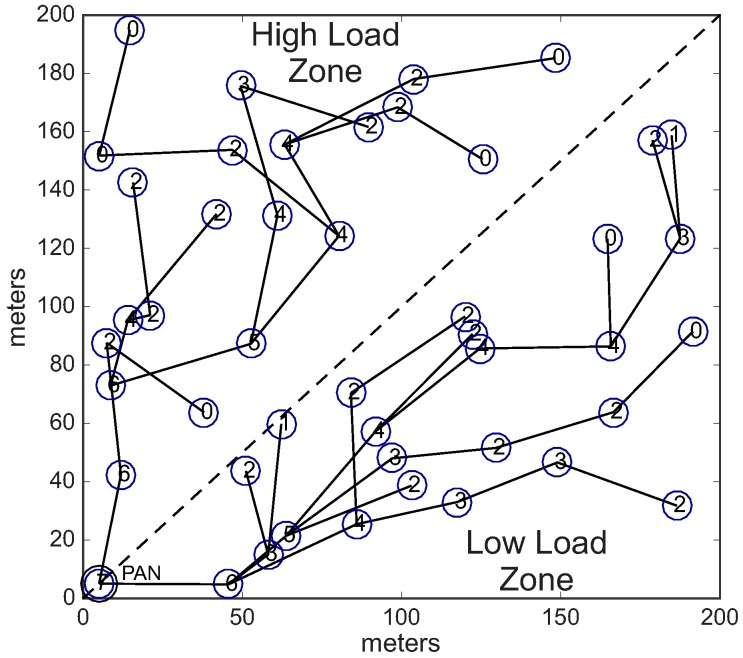
An example of Superframe Order configuration for all cluster-heads, using Nodes-SDA scheme (conditioned Scenario).

Finally, in order to highlight the differences between Load-SDA and Nodes-SDA allocation schemes, Figure 24 illustrates the superframe duration configuration (in terms of superframe order, respecting Equation (5)) provided by the Load-SDA allocation scheme, while Figure 25 illustrates the superframe duration configuration provided by the Nodes-SDA scheme for the same cluster-tree network, considering a conditioned scenario.

Figure 24 highlights that the Load-SDA scheme allocates higher values of Superframe Order for cluster-heads located in the high load zone, and lower values for cluster-heads located in the low load zone. On the other hand, the Nodes-SDA scheme allocates proportional values of Superframe Order for cluster-heads of the same depth (Figure 25), regardless of being located in low load or high load zone. Note that, as the Nodes-SDA scheme does not consider the load imposed by descendant nodes (it considers only the number of nodes), this allocation scheme may over-allocate superframe durations for cluster-heads. Importantly, this behaviour was observed for all the simulation scenarios.

Table 8 presents the average superframe order values (per depth) defined by the Load-SDA and Nodes-SDA allocation schemes, for both high load and low load zones of the conditioned cluster-tree.

In general, the Nodes-SDA scheme allocates similar superframe durations (in terms of the superframe order parameter) for cluster-heads of same depth for both zones, showing that the difference of message loads does not interfere in its allocation mechanism. In turn, the Load-SDA scheme allocates highest superframe duration values for cluster-heads located in the high load zone, while that cluster-heads located in the low load zone receive lower superframe duration values.

From this simulation assessment, it can be concluded that proportional SDA schemes can adequately allocate the required communication resources for cluster-heads (active communication periods and buffer sizes), avoiding traditional problems that occur in cluster-tree networks, such as: network congestion, high end-to-end communication delays and discarded messages due to buffer overflows. The Load-SDA scheme presents better performance for cluster-tree networks than other schemes, but it requires the knowledge of both network topology and data traffic loads. Moreover, both Load-SDA and Nodes-SDA schemes only consider cluster-tree networks where the beacon interval parameter is similar to all clusters.

## 9. Conclusions

The IEEE 802.15.4/ZigBee cluster-tree topology is one of the most suitable topologies to build wide-scale wireless sensor networks. However, these standards do not define mechanisms to adequately allocate communication resources to the cluster-heads (active communication periods and buffer sizes). Therefore, some well-known data communication problems may arise, such as: network congestion near the PAN coordinator, discarded messages due to buffer overflows and high end-to-end communication delays. In this paper, we present a set of boundary equations for IEEE 802.15.4/ZigBee cluster-tree networks (protocol, buffer and timing constraints), which provide a set of guidelines for the proper allocation of such communication resources. Within this context, we propose the use of two different proportional *Superframe Duration Allocation* (SDA) schemes: *Load-SDA* and *Nodes-SDA* schemes. The main target of these allocation schemes is to define adequate active communication periods and buffer sizes for the cluster-heads of the cluster-tree network. The Load-SDA scheme considers the message load imposed by descendant nodes to allocate superframe durations for cluster-heads, whereas the Nodes-SDA scheme considers only the number of descendant nodes of the cluster-heads.

Simulation results show that the use of adequate superframe duration allocations and buffer sizes can improve several communication metrics, such as the reduction of both the number of discarded messages due to buffer overflows and the end-to-end message communication delays. Thus, the proposed schemes can be used by network designers to build efficient cluster-tree networks in what concerns the definition of network parameters and the configuration of message streams.

As future work, we intend to add new mechanisms for the allocation schemes, such as: (a) configuration of CSMA-CA parameters to improve the message throughput; and (b) aggregation or information fusion mechanisms in order to decrease the number of messages to be transferred in congested areas of the network. Also, we intend to implement the proposed superframe allocation schemes using a real hardware testbed and to design an approach to dynamic environments (with mobile nodes).

## Figures and Tables

**Table 1:** Superframe Durations for all Clusters.

	${\mathrm{SO}}_{i}$	${\mathrm{SD}}_{i}$
$CH_{1}$	3	8 $\times SD_{min}$
$CH_{2}$	2	4 $\times SD_{min}$
$CH_{3}$	1	2 $\times SD_{min}$
$CH_{4}$	0	$SD_{min}$
$CH_{5}$	0	$SD_{min}$
$CH_{6}$	0	$SD_{min}$

**Table 2:** Timing Constraints for all Message Streams.

$S_{i}$	$R_{i}$	$P_{i}$	$R_{i}\leq P_{i}$
$S_{1}$	44.5 $\times \;SD_{min}$	60 $\times \;SD_{min}$	ok
$S_{2}$	47.5 $\times \;SD_{min}$	70 $\times \;SD_{min}$	ok
$S_{3}$	50 $\times \;SD_{min}$	60 $\times \;SD_{min}$	ok
$S_{4}$	54.5 $\times \;SD_{min}$	70 $\times \;SD_{min}$	ok
$S_{5}$	51.5 $\times \;SD_{min}$	60 $\times \;SD_{min}$	ok
$S_{6}$	54.5 $\times \;SD_{min}$	70 $\times \;SD_{min}$	ok
$S_{7}$	53.5 $\times \;SD_{min}$	60 $\times \;SD_{min}$	ok
$S_{8}$	58.5 $\times \;SD_{min}$	70 $\times \;SD_{min}$	ok
$S_{9}$	53.5 $\times \;SD_{min}$	60 $\times \;SD_{min}$	ok
$S_{10}$	58.5 $\times \;SD_{min}$	70 $\times \;SD_{min}$	ok
$S_{11}$	53 $\times \;SD_{min}$	60 $\times \;SD_{min}$	ok
$S_{12}$	56.5 $\times \;SD_{min}$	70 $\times \;SD_{min}$	ok

**Table 3:** Summarising the different Simulation Scenarios.

Features	Unconditioned Scenario	Conditioned Scenario
Maximum number of child nodes (per cluster)	6 nodes	6 nodes
Maximum number of CCH for PAN coordinator	2 nodes	2 nodes
Maximum number of CCH (other CHs)	2 nodes	3 nodes
Set of data rates	0.05 and 0.01 pkts/s	0.05 and 0.01 pkts/s
Data rate distribution in the environment	randomly distributed	two well-defined zones

**Table 4:** Simulation Configuration.

Definition	Standard Value
Radio model	Chipcon CC2420
Initial energy (per node)	18.720 Joules
Simulation time (each experiment)	110.000 s
*aBaseSlotDuration*	60
*aNumSuperframeSlots*	16
*aUnitBackoffPeriod*	20
*macMinBE*	3
*macMaxBE*	5
*macMaxCSMABackoffs*	4
*macMaxFrameRetries*	3

**Table 5:** Information about the network formation.

Information	Unconditioned Scenario	Conditioned Scenario
Average number of clusters	45	47
Average maximum depth	9	8
Average number of children per cluster	4	4

**Table 6:** Buffer size for cluster-heads (per depth).

	Unconditioned Scenario			Conditioned Scenario		
Depths	Load/Nodes-SDA	SOA-SDA	STA-SDA	Load/Nodes-SDA	SOA-SDA	STA-SDA
Depth 1	98	200	200	98	200	200
Depth 2	47	200	200	39	200	200
Depth 3	24	200	200	19	200	200
Depth 4	20	200	200	15	200	200
Depth 5	17	200	200	12	200	200
Depth 6	12	200	200	9	200	200
Depth 7	7	200	200	6	200	200
Depth 8	5	200	200	6	200	200
Depth 9	5	200	200	7	200	200
Depth 10	2	200	200	4	200	200

**Table 7:** Average Superframe Order (SO) parameter values for cluster-heads (per depth).

	Unconditioned Scenario				Conditioned Scenario			
Depths	Load	Nodes	SOA	STD	Load	Nodes	SOA	STD
Depth 0	6	7	6	3	6	7	7	3
Depth 1	5	6	5	3	5	6	5	3
Depth 2	4	5	3	3	3	4	2	3
Depth 3	3	3	1	3	2	3	1	3
Depth 4	2	3	1	3	2	3	1	3
Depth 5	2	3	1	3	2	2	0	3
Depth 6	2	2	1	3	1	2	0	3
Depth 7	1	2	0	3	1	1	0	3
Depth 8	1	1	0	3	1	1	0	3
Depth 9	1	1	0	3	1	1	0	3
Depth 10	0	0	0	3	1	1	0	3

**Table 8:** Average Superframe Order values (per depth) for both the high load and low load zones (conditioned Scenario).

	Load-SDA Scheme		Nodes-SDA Scheme	
Depths	High Load Branch	Low Load Branch	High Load Branch	Low Load Branch
Depth 1	6	4	6	6
Depth 2	4	2	4	4
Depth 3	3	1	3	3
Depth 4	3	1	3	2
Depth 5	3	1	3	2
Depth 6	2	1	2	2
Depth 7	2	1	2	1
Depth 8	1	1	1	1
Depth 9	1	-	1	-
Depth 10	1	-	1	-

## Abbreviations

The following acronyms are used in this manuscript:

*α*	*aBaseSuperframeDuration*
$\delta_{i}$	Enough time to transmit the message $M_{i}^{k}$
*η*	Number of messages generated by message streams located below of $CH_{j}$
$\gamma_{i}$	Initial delay for message stream $S_{i}$
$\sigma_{i}$	Time interval immediately lower than the transmission time for one message $M_{i}^{k}$
Θ	Interference imposed by highest priority message streams
*b*	Maximum number of backoffs
$M_{i}^{k}$	K-th message of Message Stream i generated by node $N_{n}$
$N_{nodes}$	Number of sensor nodes
$n_{CH}$	Number of child cluster-heads
$N_{CH}$	Number of cluster-heads
$n_{l}$	Number of leaf child nodes
$P_{c}$	Probability of a node to perform two consecutive CCA analysis
$P_{co}$	Collision probability of two nodes
$P_{s}$	Probability of a node to perform a CCA with the maximum backoff number
*q*	Probability that a node transmits a single data at any time of the CAP
*r*	Average backoff number
$R_{i}$	Worst-case response time for message stream $S_{i}$
S	Set of Message Streams
$S_{below}^{j}$	Set of message streams located in the descendant nodes of $CH_{j}$
$S_{i}$	Message Stream *i*
$T_{BO}$	Average backoff time
$T_{BOL}$	Backoff period length
$T_{BOT}$	Total backoff time
$T_{radio\_transition}$	Time duration that the radio takes to switch between the different operation modes
$T_{TXD}$	Total transmission time for a single data frame
*U*	Total Effective Utilisation
*W*	Local worst-case response time
